# The association between eight complete blood count-derived inflammatory markers and muscle health

**DOI:** 10.3389/fnut.2025.1498757

**Published:** 2025-02-03

**Authors:** Jingyuan Zhang, Yuqi Wang, Heng Liu, Zhuolin Lei, Shouquan Cheng, Hong Cao

**Affiliations:** ^1^Department of Traumatic Orthopedics, Renmin Hospital, Hubei University of Medicine, Shiyan, China; ^2^Department of Traumatic Orthopedics, Weifang Yidu Central Hospital, Shiyan, China; ^3^Department of Urology, Renmin Hospital, Hubei University of Medicine, Shiyan, Hubei, China

**Keywords:** sarcopenia, muscle mass, inflammation, indicators, NHANES

## Abstract

**Background:**

Most studies have evaluated sarcopenia and muscle health solely based on muscle mass. This study comprehensively examined the associations between eight inflammatory indicators and muscle mass and strength, with the aim of identifying an indicator capable of evaluating muscle health across multiple dimensions.

**Methods:**

This study included 10,440 participants from the National Health and Nutrition Examination Survey (NHANES, 2011–2018) and 5,384 participants from NHANES (2011–2014). Multivariate logistic regression, smooth curve fitting, restricted cubic spline (RCS) analysis, subgroup analysis, and Spearman's correlation were used to comprehensively assess the associations between the eight inflammatory indicators and muscle mass and strength. Receiver operating characteristic (ROC) curves were used to compare the predictive abilities of the different indices for low muscle mass and muscle strength. Additionally, NHANES data were cross-validated with data from 554 patients at our hospital to evaluate the ability of the systemic immune inflammatory index (SII) to distinguish between low muscle mass and strength.

**Results:**

After controlling for all potential confounding factors, multiple logistic regression analysis revealed that apart from the platelet-to-lymphocyte ratio (PLR), monocyte-to-lymphocyte ratio (MLR), and derived NLR (dNLR), the neutrophil-to-monocyte-plus-lymphocyte ratio (NMLR), neutrophil-to-lymphocyte ratio (NLR), SII, systemic inflammation response index (SIRI), and pan-immune-inflammation value (PIV) were significantly negatively correlated with muscle mass and strength. However, NMLR and NLR were significantly associated with changes in muscle mass only in Q4 (*P* < 0.05). In the stratified analysis by body mass index (BMI), only the SII, NLR, and NMLR were unaffected by BMI. In the cross-validation, the predictive performance of the SII for low muscle mass [area under the curve (AUC) = 0.699, 0.677, and 0.685] and low muscle strength (AUC = 0.857, 0.849, and 0.840) demonstrated a good reference value. RCS and smooth curve fitting analyses indicated that most inflammatory markers were linearly correlated with muscle health (*P* < 0.05).

**Conclusion:**

Compared with other inflammatory markers (e.g., PIV and dNLR), the SII demonstrated a more robust predictive ability, was less influence by covariates, and exhibited high generalization performance in internal and external validation. SII may be crucial in identifying “hidden sarcopenia” and the early stages of muscle functional decline.

## Graphical Abstract

Normal muscle mass but low muscle strength may suggest cryptogenic sarcopenia or muscle dysfunction. 
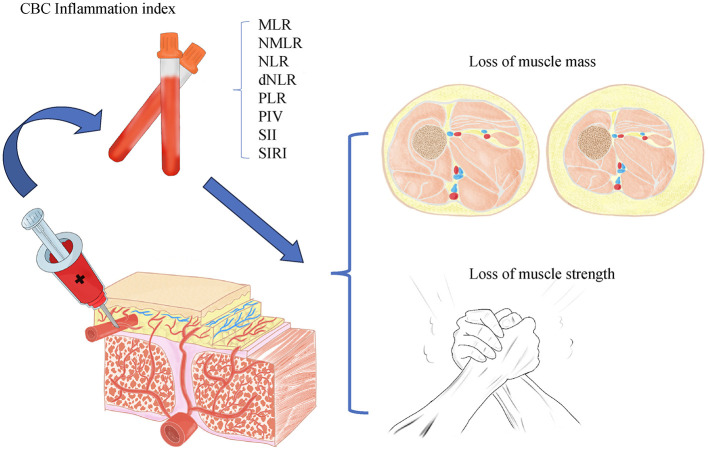

## 1 Introduction

Short-term inflammation can attract immune cells, such as white blood cells, to promote tissue healing and recovery; however, persistent inflammatory stimulation is the root cause of many chronic diseases. Complete blood count (CBC) is a routine laboratory test that evaluates various blood components. Inflammatory markers derived from this test can provide value beyond a single indicator for predicting disease status and prognosis by integrating different components of the blood (1–3).

For example, studies have reported that the systemic immune inflammatory index (SII) and prolactin (PLR) test show superior accuracy and stability in predicting postmenopausal osteoporosis (4). The neutrophil-to-lymphocyte ratio (NLR) is also an effective biomarker to predict the severity of atherosclerosis, and its prognostic value is no less than that of traditional inflammatory markers. The pan-immune-inflammation value (PIV) reflects uncontrolled inflammation during the development and progression of heart failure and can effectively predict the prognosis of patients with acute heart failure (5, 6). Furthermore, Zhang et al. (7) reported that the SII is significantly more accurate than c-reactive protein (CRP) in predicting coronary heart disease. Wang et al. (8) found that NLR and PLR can effectively predict the development of insulin resistance and are important to predict the deterioration of renal function in patients with diabetic kidney disease (DKD). Especially in patients with T2DM, the predictive accuracy of the NLR is significantly higher than that of CRP and erythrocyte sedimentation rate. The systemic inflammation response index (SIRI) is widely used to evaluate the prognosis of various tumors, such as head and neck cancer and gastric cancer (9, 10). SIRI can also more accurately predict the 5- and 10-year survival rates of patients with breast cancer than tumor, node, and metastasis staging alone. The change in SIRI from baseline to 4 weeks after surgery is closely related to the survival rate of patients with breast cancer (11). In addition, the economy and portability of routine blood tests indicate that these inflammatory markers have application values far exceeding those of other markers in clinical practice and correlation prediction.

Muscle health is closely linked to quality of life and disease prevention. As the body's primary calcium reservoir, muscles store significant amounts of calcium ions, which help prevent osteoporosis (12). Additionally, muscles of sufficient quality and strength can protect the bones during external impacts. However, studies have only evaluated muscle health from the perspective of muscle mass and used the decrease in muscle mass as the only criterion for evaluating sarcopenia (13, 14). A decline in muscle strength not only affects the functional activity ability of the elderly (climbing stairs, walking, etc.), but is also associated with a variety of adverse outcomes (such as decreased self-care ability, increased risk of falls, etc.). In a cohort of Korean patients with non-alcoholic fatty liver disease, muscle strength outperformed muscle mass in predicting advanced fibrosis (15, 16). A recent study also identified slow gait speed as an independent risk factor for increased all-cause mortality; therefore, muscle strength may have a better predictive value for disease than muscle mass. According to the latest epidemiological studies, as the problem of population aging becomes more serious, the incidence of muscle diseases among the elderly increases every year (17, 18). Muscle diseases, such as sarcopenia, are associated with poor prognosis for multiple chronic diseases (such as diabetes, heart failure, and COPD), which impose a huge burden on less-developed countries (19–21); however, the impact on developed countries such as the United States cannot be ignored.

To our knowledge, existing studies have mostly focused on the association between muscle mass and a single inflammatory marker, and there is a lack of studies that have systematically evaluated the crosstalk between multiple inflammatory markers and muscle mass and strength (22). Although the effect of muscle mass on health has received extensive attention in recent years, the importance of muscle strength as an independent predictor is often overlooked. We, therefore, aimed to evaluate muscle mass and strength as a whole for the first time to comprehensively reveal the associations between multiple CBC-derived inflammatory markers and muscle health. In addition, we determined which inflammatory markers can effectively reflect muscle mass and muscle strength simultaneously, and further compare the comprehensive ability of different markers in assessing muscle health.

## 2 Methods

### 2.1 Study population and participants

**Figure 1:**
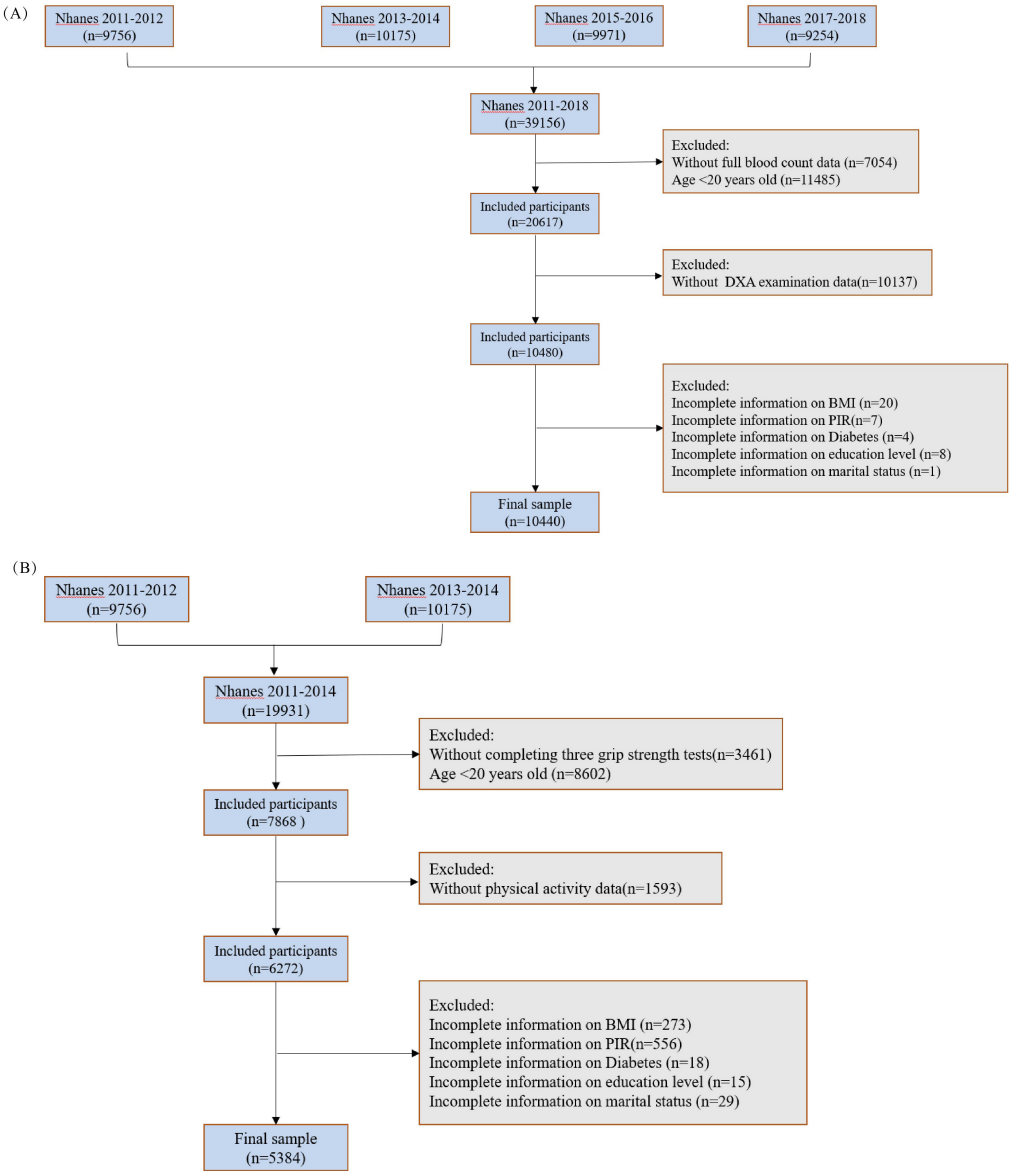
Inclusion and exclusion criteria of the study population. **(A)** Muscle mass; **(B)** muscle strength.

The National Health and Nutrition Examination Survey (NHANES) is a large cross-sectional survey that uses a multistage, multistratum sampling approach to ensure that the sample is representative of the general United States population. The survey was conducted every 2 years and is updated regularly. A total of 39,156 participants were screened between 2011 and 2018. After excluding 7,054 respondents with missing CBC-related data, 11,485 respondents under the age of 20 years, 5,094 respondents with missing grip strength test results, 10,137 respondents with missing dual-energy X-ray absorptiometry data, and 40 respondents with missing covariates, 10,440 respondents from 2011 to 2018 were included in the muscle mass study. The inclusion criteria for the muscle strength study were as follows: (1) participants aged 20 years or older; (2) completion of all three grip strength tests; (3) no missing data for CBC; and (4) no missing covariates. After excluding 14,547 respondents who did not meet the criteria from 19,931 participants between 2011 and 2014, 5,384 participants were finally included (Figure 1).

### 2.2 Ethical statement

This study strictly adhered to the ethical standards of the Helsinki Declaration issued in 1964 and its subsequent revisions and was approved by the National Health Statistics Research Ethics Review Committee; participant personal information will be kept confidential, used only for research purposes, and presented anonymously.

### 2.3 Study variables and covariates

Based on previous literature, this study used multiple variables to construct a relative handgrip strength index (HGS/BMI) and appendicular skeletal muscle mass index (ASM/BMI). Handgrip strength (HGS) was determined using three grip strength measurements with the participant's elbow fully extended. The relative strength index was calculated by dividing the HGS of the dominant hand by BMI (23, 24). For participants with equal grip strength in both hands, the average grip strength of both hands was used. In this study, both muscle quality and strength indices were categorized into four levels according to quartiles: Q1 (< 25%), Q2 (25–50%), Q3 (50–75%), and Q4 (>75%), enabling a more detailed analysis of the data. After grouping by gender, the quartiles of the relative strength index were calculated separately for each gender, with Q1 defined as low muscle strength (LMS) (25). ASM was calculated as the sum of the appendicular skeletal muscle masses (kg), and the muscle mass index was derived by dividing ASM by body mass index BMI (kg/m^2^). According to guidelines published by the Foundation for the National Institutes of Health, respondents with a muscle mass index < 0.789 for men and < 0.512 for women were classified as having LMS and defined as having sarcopenia (26–29). This study combined these two indices to provide a more comprehensive assessment of muscle health.

Since platelets cannot be expressed as a percentage, all CBC-derived inflammatory markers were calculated using the absolute counts of CBC parameters (10^3^ cells/μL). This study primarily included eight indicators calculated using the following formulas (30):


$$\begin{array}{l} MLR=\frac{monocytes}{lymphocytes}\\ NMLR=\frac{\left(monocytes\;+\;neutrophils\right)}{lymphocytes}\\ NLR\;=\frac{neutrophils}{lymphocytes}\\ dNLR\;=\frac{neutrophils}{\left(white\;cell\;count\;-\;lymphocytes\right)}\\ PLR\;=\frac{platelets}{lymphocytes}\\ PIV\;=\frac{neutrophils\;\times \;monocytes\;\times \;platelets}{lymphocytes}\\ SII\;=\frac{platelets\;\times \;neutrophils}{lymphocytes}\\ SIRI\;=\frac{neutrophils\;\times \;monocytes}{lymphocytes} \end{array}$$

To meet the performance requirements of the model, covariates with minimal missing data and relevance to both independent and dependent variables were selected to reduce errors and enhance explanatory power. Covariates were categorized into continuous and categorical variables. Continuous variables included age and income-to-poverty ratio, while categorical variables included sex, race, marital status, smoking habits, educational level, history of diabetes, and moderate physical activity.

### 2.4 Statistical analysis

Linear relationships between CBC-derived inflammatory markers and muscle mass and strength were assessed using multiple linear regression analysis, whereas non-linear relationships were explored using smoothing curve fitting and threshold effect analysis. Non-linear associations among low muscle mass, LMS, and inflammatory markers were analyzed using restricted cubic spline (RCS) curves. A recursive algorithm was employed to scan the variable range and the cut.tab threshold effect function in the ggrcs and rcssci packages was used to accurately identify potential inflection points. Receiver operating characteristic (ROC) curve analysis was conducted to determine the best indicator to predict the risk of low muscle mass and strength. Spearman's correlation analysis was used to evaluate the association between the CBC-derived inflammatory markers and these outcomes.

To thoroughly explore the relationships between the study variables and outcomes, multi-model regression was performed by gradually adding adjusted variables to construct three regression models of varying complexity. Model 1 included no adjustments, while Model 2 adjusted for demographic variables (age, sex, and race) to control for basic demographic differences, and preliminarily assessed the associations between inflammatory markers and muscle health without the influence of other confounding factors. Model 3 incorporated additional variables, including income-to-poverty ratio (IPR), BMI, education level, marital status, and lifestyle and health behaviors (e.g., smoking, diabetes, and moderate physical activity), to fully control for confounders and examine the independent effects of CBC-derived inflammatory markers on muscle mass (31, 32).

To enhance the generalizability of the model and avoid overfitting, the inflammatory markers were stratified into quartiles to minimize the influence of outliers. In addition, external datasets were used for cross-validation to ensure the robustness of the validation model. Hierarchical smooth curve fitting was used to visually demonstrate the confounding effects of BMI on the results.

## 3 Result

### 3.1 Baseline characteristics

The baseline characteristics of the respondents were stratified by quartiles of muscle mass index and strength index (Supplementary Tables S1, S2). Participants in the highest quartile of muscle mass index had a higher proportion of male and middle-aged individuals, an increasing poverty-to-income ratio, a greater proportion of non-Hispanic Black participants, and a higher prevalence of smoking than those in the lowest quartile. Conversely, participants in the lowest quartile exhibited a lower prevalence of diabetes mellitus and reduced absolute counts of white blood cells, platelets, monocytes, lymphocytes, and neutrophils than those in the highest quartile.

The quartile cutoffs for the strength indices were 0.97, 1.27, 1.61, and 3.51 for Q1–4, respectively. Muscle strength increased with decreasing BMI, age, and diabetes prevalence, and was associated with higher proportions of non-Hispanic Black people and married or partnered individuals. Similar to the muscle mass index, participants in the highest quartile of the strength index had lower absolute counts of white blood cells, platelets, monocytes, lymphocytes, and neutrophils, and higher household income and education levels.

### 3.2 CBC-derived inflammatory markers and muscle mass and strength

#### 3.2.1 Quantitative association with muscle mass

This study analyzed the relationship between CBC-derived inflammatory markers and muscle mass using weighted linear regression models. To explore the relationships between the variables and outcomes comprehensively, the models were gradually adjusted, resulting in three regression models with varying levels of complexity. After adjusting for demographic variables in Model 2, most inflammatory marker effect sizes (β) decreased, and the effect of PLR as a continuous variable was no longer significant. Further comprehensive adjustment for covariates in Model 3 eliminated the independent effects of MLR, NMLR, NLR, and PLR as continuous variables (β = 0.00, *P* = 0.999). When muscle mass was grouped into quartiles, a significant negative correlation among NMLR, NLR, and muscle mass was observed in the highest quartile (Q4). By contrast, the effects of MLR and PLR disappeared entirely. Additionally, the dNLR, SIRI, and PIV demonstrated significant negative correlations as continuous variables; however, in the quartile group analysis, the correlations for these markers in the Q2 group did not reach statistical significance.

Notably, the SII maintained a significant negative correlation with muscle mass (*P* < 0.001) when analyzed as both a continuous and categorical variable, indicating a robust independent predictive effect (Supplementary Table S3). Specifically, the muscle mass index decreased by 0.01 and 0.02 units for each unit increase in NMLR and NLR, respectively, when NMLR and NLR were below thresholds of 2.88 and 2.63 (β = −0.01, 95% CI: −0.02 to −0.00; β = −0.02, 95% CI: −0.02 to −0.01). Similarly, the muscle mass index decreased by 0.006 and 0.026 units for each unit increase in SII and SIRI, respectively, when SII and SIRI were below thresholds of 608.20 and 1.77(β = −0.01, 95% CI: −0.01 to −0.00; β = −0.03, 95% CI: −0.04 to −0.01). Furthermore, when PIV was below 353.85, each unit increase in PIV was associated with a 0.03 unit decrease in the muscle mass index (β = −0.17, 95% CI: −0.28 to −0.06).

In summary, NMLR, NLR, dNLR, SII, SIRI, and PIV were negatively correlated with muscle mass. Among these markers, NMLR and NLR showed significant correlations only in the highest quartile group (Q4), whereas dNLR, SIRI, and PIV were not significant in the Q2 group. In contrast, the SII consistently demonstrated a robust negative correlation across all quartiles.

#### 3.2.2 Quantitative associations with muscle strength

Three models were constructed using the same methodology to investigate the relationship between the CBC-derived inflammatory indicators and muscle strength. After full adjustment for covariates, no subgroups of the PLR or dNLR showed significant associations, whereas the NLR, MLR, NMLR, SII, SIRI, and PIV were significantly negatively correlated with muscle strength. In continuous variable analysis, the independent effects of MLR, NLR, dNLR, and PLR were not significant after full adjustment; however, the highest quartile (Q4) of NLR and MLR showed significant negative correlations.

Further quartile analysis revealed that the effects of MLR, SIRI, and PIV were not significant in the Q2 and Q3 groups, but were significantly negatively correlated with muscle strength in the Q4 group (Supplementary Table S4). Meanwhile, NMLR and NLR were significantly negatively correlated with muscle strength in the Q2, Q3, and Q4 groups, with the Q4 group showing the most substantial effect (NMLR: β = −0.09, 95% CI: −0.13 to −0.04, *P* < 0.001; NLR: β = −0.09, 95% CI: −0.13 to −0.04, *P* < 0.001). Notably, although SII did not reach statistical significance in the Q2 group, it demonstrated a significant negative correlation in both the Q3 and Q4 groups, with the Q4 group showing the most pronounced effect (β = −0.07, 95% CI: −0.11 to −0.03, *P* = 0.002).

As the NLR, MLR, NMLR, SII, SIRI, and PIV increased, the muscle strength index progressively decreased, with Q4 showing the strongest effect. Specifically, each 1-unit increase in the NLR was significantly associated with a 0.09 decrease in the muscle strength index. Similarly, each 1-unit increase in the MLR was significantly associated with a 0.01 decrease, and each 1-unit increase in the NMLR was significantly associated with a 0.09 decrease in the muscle strength index. Furthermore, each 1-unit increase in SII, SIRI, and PIV was significantly associated with a 0.07 decrease in the muscle strength index.

In summary, NLR, MLR, NMLR, SII, SIRI, and PIV were negatively correlated with changes in muscle strength. However, NMLR and NLR showed significant effects only in the Q4 group.

#### 3.2.3 Correlation analysis of CBC-derived inflammatory markers with sarcopenia and LMS

**Figure 2:**
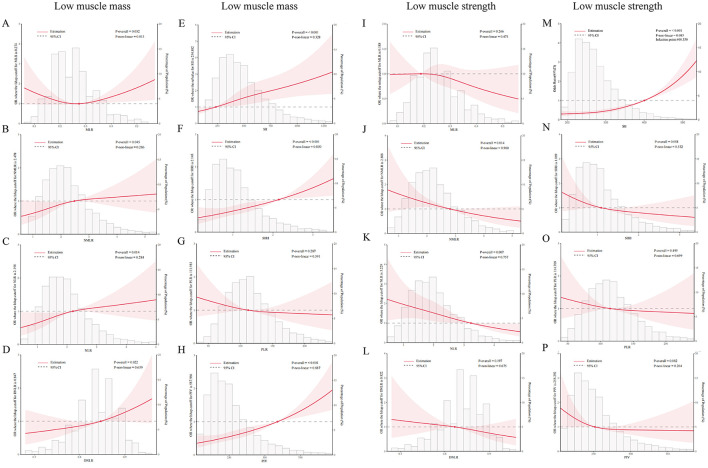
Correlations between inflammatory markers and low muscle mass (sarcopenia) and low muscle strength (LMS) were analyzed using RCS curves. **(A–H)** Associations between MLR, NMLR, NLR, dNLR, SII, SIRI, PLR, and PIV and the risk of low muscle mass. **(I–P)** Associations between the markers and risk of LMS. The red dots in the figures indicate key turning points, while the red shaded areas represent the 95% confidence intervals.

This study also used RCS curves to analyze the association between eight inflammatory markers, low muscle mass (sarcopenia), and LMS (Figure 2). After adjusting for covariates including sex, age, race, PIR, education level, marital status, smoking habits, diabetes, and BMI, the models revealed that the associations between various inflammatory markers and muscle health differed to some extent.

In the analysis of low muscle mass, only MLR exhibited a significant non-linear association (*P* = 0.032 and P-nonlinea*r* = 0.013, respectively). Other inflammatory markers, including the NLR, dNLR, PIV, SII, and SIRI, showed statistically significant positive linear correlations with low muscle mass (*P* < 0.05).

In the analysis of LMS, the results indicated a negative linear correlation between the NMLR, NLR, and LMS (*P* < 0.05). Additionally, the SII demonstrated a significant positive correlation with LMS. The remaining indicators did not show significant correlations.

### 3.3 Non-linear and saturation effects

Smooth curve fitting and piecewise linear regression models were used to explore the non-linear relationships and saturation effects between the eight inflammatory markers, muscle mass, and strength. The results showed a non-linear relationship between NMLR, dNLR, PLR, SII, SIRI, PIV, and muscle mass (Supplementary Figure S1), and their key inflection points were 2.88, 74.43, 608.21, 1.77, and 355.40 (Supplementary Table S5). Log-likelihood ratio tests confirmed that these non-linear relationships were statistically significant (*P* < 0.05). For inflammatory markers, such as NLR, dNLR, SII, SIRI, and PIV, values below the inflection point showed a significant negative correlation with muscle mass. In contrast, the values above the threshold were not statistically significant.

In the piecewise linear regression analysis of inflammatory markers and muscle strength, non-linear relationships were observed between NMLR, NLR, SII, SIRI, and PIV (log-likelihood ratio < 0.05). No significant non-linear correlations were identified between the remaining markers and muscle mass.

### 3.4 Subgroup analysis

To further minimize bias in the results caused by confounding factors, we conducted a stratified analysis of the data based on age, sex, race, education level, IPR, diabetes status, smoking status, and activity level. After adjusting for all covariates, smoking status (interaction, *P* < 0.05) was the only stratification variable that significantly influenced the relationship between CBC-derived inflammatory markers, and muscle mass and strength. No significant interactions were observed for other variables such as age, sex, or race (Supplementary Figure S2).

Multiple inflammatory markers showed stronger negative associations with muscle mass and strength among non-smoking respondents. In the subgroup analysis of muscle mass, the negative correlations between muscle mass and NMLR, NLR, SII, SIRI, and PIV were more pronounced in the non-smoking group (*P* < 0.001). Similarly, in the subgroup analysis of muscle strength, the SII, SIRI, and PIV exhibited significant negative correlations with muscle strength only in non-smokers, whereas these associations were not significant in smokers.

**Figure 3:**
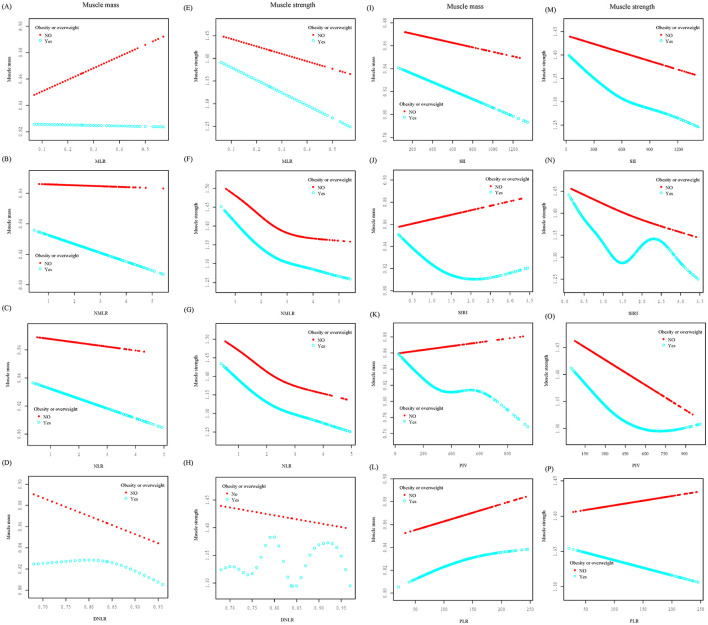
Smoothed curve fitting was conducted stratified by obesity or overweight status. The relationships between various inflammatory markers and muscle mass **(A–D, I–L)** and muscle strength **(E–H, M–P)** were analyzed. In the figures, the red curve represents non-obese/non-overweight individuals, while the blue curve represents obese/overweight individuals.

Additionally, stratified smoothing curves were fitted based on respondents' overweight or obese status (BMI ≥ 25) (Figure 3). In the muscle strength group, the curves for the SIRI and MLR in overweight or obese individuals (blue curve) showed significant fluctuations, indicating that being overweight or obese had a notable impact on these two markers. In the muscle mass group, SIRI and PIV exhibited a positive correlation in non-overweight or non-obese individuals (red curve), in contrast to the negative correlations observed in overweight or obese individuals.

In summary, the negative correlations of multiple inflammatory markers with muscle mass and strength were more pronounced in the non-smoking population. The stratified smoothing curve fitting results indicated that only SII, NLR, and NMLR demonstrated linear or non-linear associations with muscle mass and strength, unaffected by BMI status.

### 3.5 ROC analysis

**Figure 4:**
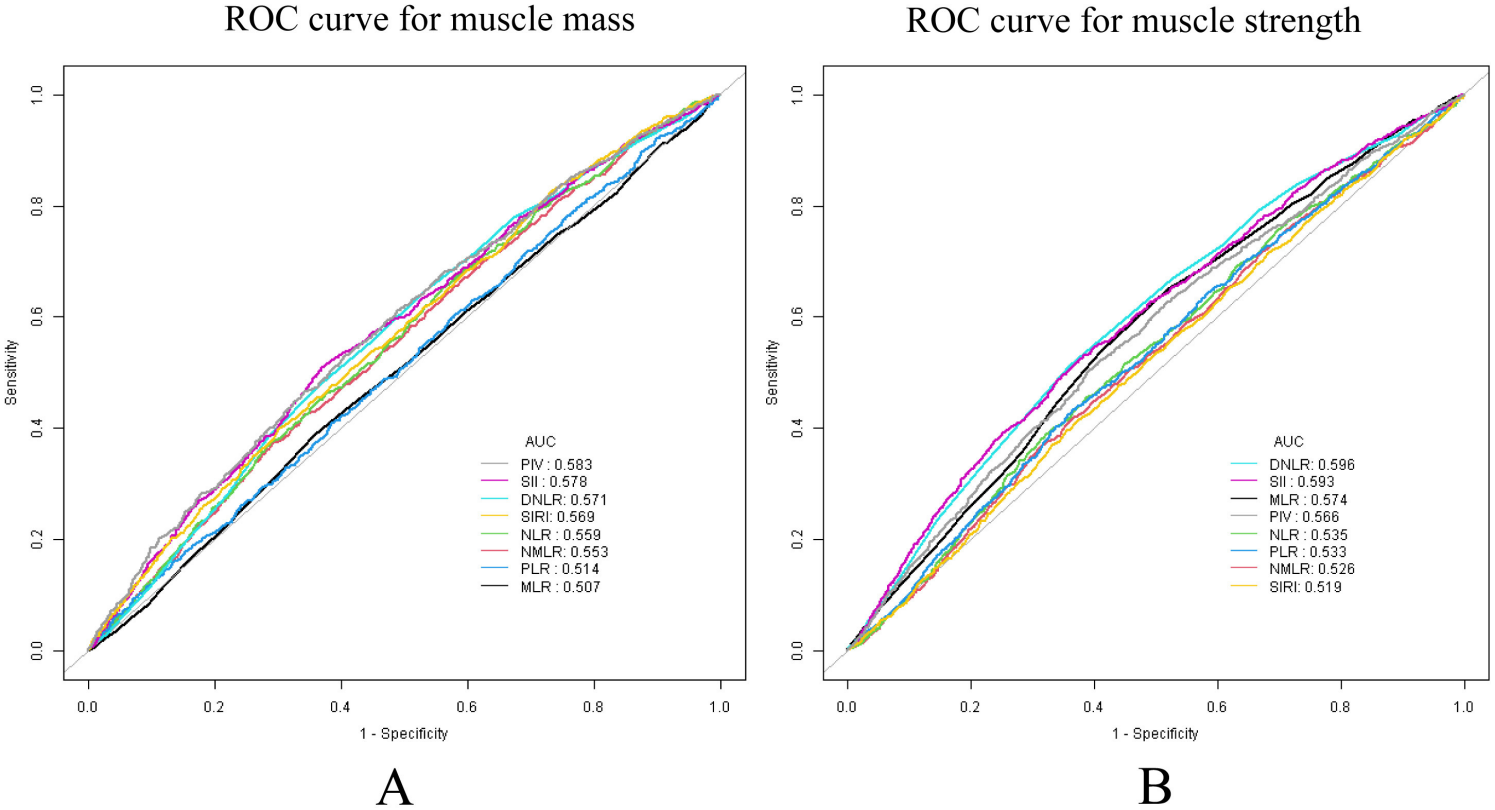
ROC analysis, used to evaluate the predictive accuracy of SII and other inflammatory markers for low muscle mass and strength. **(A)** Low muscle mass; **(B)** low muscle strength.

The diagnostic performances of the eight inflammatory markers for low muscle mass and strength were investigated using ROC curves (Figure 4). The performance of the SII was relatively balanced for both outcomes, with AUC values of 0.578 for muscle mass and 0.593 for muscle strength (Supplementary Table S6). Although the SII did not have the highest AUC value, it demonstrated a stable discriminative ability for both indicators. Notably, while the dNLR exhibited the strongest discriminative ability for muscle strength (AUC = 0.596), its performance was highly susceptible to the influence of other covariates in a multivariate context.

### 3.6 Correlation between markers of inflammation and muscle mass or strength

This study also explored the correlation between inflammation-related indicators and blood cell counts from 2011 to 2018 and 2011 to 2014 (Supplementary Figure S3). The analysis revealed the strongest negative correlation between PLR and LYM (*r* = −0.71), whereas SIRI and PIV exhibited a strong positive correlation (*r* = 0.93). Additionally, a strong positive correlation was observed between the SII and PIV (*r* = 0.86).

### 3.7 Cross-validation of SII

This study also used NHANES data and data from 554 patients at our hospital for cross-validation to assess the ability of the SII to distinguish between low muscle mass and LMS. To enhance the model, both univariate and multivariate regression analyses were conducted, incorporating confounding factors, such as age, sex, race, education level, smoking status, and diabetes. Participants included in the internal validation were randomly assigned to a training set and a validation set in a 7:3 ratio based on the NHANES data. The external validation set consisted of 554 participants randomly selected from our hospital between 2021 and 2024, who adhered to the same inclusion and exclusion criteria. After the evaluation, the most influential variables (age, sex, diabetes status, activity level, and SII) were retained in the final model (Supplementary Table S7).

In the low muscle mass group, the model demonstrated relatively consistent AUC values across the training, validation, and external validation sets. The AUC value for the external dataset (AUC = 0.699, 95% CI: 0.694–0.834) was higher than those for the training (AUC = 0.677, 95% CI: 0.654–0.799) and validation (AUC = 0.685, 95% CI: 0.676–0.802) sets. Calibration curve analysis indicated that the predicted probabilities for the external validation set in the low-to-moderate risk range were highly consistent with the observed outcomes (Supplementary Figures S4–S7).

In the LMS group, the results indicated that the training set achieved an AUC of 0.849 (95% CI: 0.827–0.904), while the internal validation set demonstrated similar performance with an AUC of 0.840 (95% CI: 0.824–0.914). The external validation set also exhibited a robust predictive ability, with an AUC of 0.857 (95% CI: 0.850–0.942). Additionally, the calibration curve for the external validation set showed a good fit in the moderate risk range of 0.2–0.8.

## 4 Discussion

This study included 15,824 patients from the NHANES database for association analysis and internal validation model construction, and 554 patients from our hospital for external validation. The results showed that in addition to PLR and MLR, NMLR, NLR, DNLR, SII, SIRI, and PIV were significantly negatively correlated with muscle mass. However, NMLR, NLR, DNLR, SIRI, and PIV were not significantly different among the four groups, with only SII consistently showing a robust negative correlation. Among the eight indices, NLR, MLR, NMLR, SII, SIRI, and PIV were negatively correlated with changes in muscle strength, although NMLR and NLR showed significant effects only in the Q4 group. Further analysis revealed a negative linear correlation between NMLR and NLR and LMS, whereas the SII was significantly positively correlated with LMS (*P* < 0.05). The remaining indices did not exhibit significant correlations. Notably, the SII demonstrated a stable discriminative ability for both muscle mass and LMS, with minimal susceptibility to other covariates. The consistency between the internal and external validations underscores that the SII has a reliable predictive accuracy and overall stability across different patient populations.

Skeletal muscle is essential to maintain normal physiological functions of the human body. Muscle mass and strength are commonly used to evaluate skeletal muscle function. Numerous studies have shown a clear relationship between systemic inflammatory markers and skeletal muscle function (33, 34); however, current research primarily focuses on the association between muscle mass and a single inflammatory marker. There is a lack of studies that systematically evaluate the interaction between multiple inflammatory markers and both muscle mass and strength (22). Our study is the first to consider muscle strength and mass together and simultaneously assess their correlation with eight inflammatory markers: MLR, NMLR, NLR, dNLR, PLR, PIV, SII, and SIRI.

Several other disease models have used the NLR and SII as important indicators for disease prediction. Wang et al. (35) demonstrated that the NLR is the best predictor of stroke-associated pneumonia (SPA) and poor prognosis in patients with cerebral hemorrhage, and can be used to identify severe SPA early. Xie et al. (36) found that elevated SII levels are associated with hepatic fatty degeneration. To further validate the continuity and independence of the SII and NLR in assessing muscle health and to exclude the influence of BMI on prediction efficiency, we performed stratified smooth curve fitting according to BMI status. Among the eight inflammatory indicators, only the linear or non-linear correlations between the SII, NLR, and NMLR and muscle mass and strength were unaffected by BMI. However, the independent effects of NLR and NMLR were no longer significant in the weighted linear regression model (Model 3) after comprehensive adjustment for the variables. Ultimately, the SII has been proven to be an independent marker to assess muscle mass and strength. This finding is supported by a study by Shi et al. (37), who reported that the SII independently increased the risk of low muscle mass. However, they did not observe any relationship between SII and muscle strength, which remains a limitation of most studies. Notably, in our stratified analysis, smoking status altered the correlation between multiple inflammatory markers, muscle mass, and strength. Overall, the negative correlations between muscle mass and strength and SII, SIRI, and PIV were more significant in non-smokers (*P* < 0.001). A potential explanation for this finding is that smoking contributes to skeletal muscle dysfunction. Degens et al. (38) suggested that the reduced contractile endurance of skeletal muscle may result from the interaction of carbon monoxide with hemoglobin, myoglobin, and components of the respiratory chain, leading to impaired oxygen delivery to mitochondria and reduced mitochondrial ATP production. Chan et al. (39) also demonstrated that smoking exposure not only prevents the activation of muscle stem cells, but also induces muscle inflammation. In particular, the recruitment of F4/80+ monocytes to the injury site was amplified and the expression of pro-inflammatory cytokines was enhanced. These findings align with our, in which the biological activities of inflammatory cells caused by the inflammatory response led to changes in the inflammatory indicators included in this study.

Muscle health involves a series of biochemical reactions in the body, which are inevitably accompanied by changes in inflammatory factors. SII was calculated based on the levels of platelets, neutrophils, and lymphocytes in the blood. Studies on the pathogenic mechanisms of sarcopenia and low muscle density have shown that interleukin-6 (IL-6) and TNF-α play key roles (40). Elevated levels of both cytokines lead to neutrophil expansion, which is reflected by the upregulation of SII. The proposed mechanism is that neutrophil-related inflammatory factors are upregulated, resulting in an increase in the SII, which indirectly indicates that the patient's muscle health is compromised. Lymphocytes are primarily involved in maintaining cellular homeostasis. In an immunohistochemistry and CT scan study, the numbers of CD8+ T cells and total T lymphocytes were positively correlated with muscle mass (41). This explains why an increase in lymphocyte count is associated with a decrease in the SII and an increase in muscle mass. However, the relationship between platelets, muscle strength, and muscle mass has not been extensively studied. Neutrophils rapidly accumulate within 24 h of muscle injury, releasing reactive oxygen species and elastase to clear necrotic tissue (42). Neutrophils peak at 48 h, releasing IL-6 and TNF-α to recruit monocytes and macrophages (43). Platelets play a crucial role in the recruitment of neutrophils to injured skeletal muscles.

Finally, although traditional views often regard low muscle mass as the core problem of sarcopenia, the consideration of both muscle mass and strength is essential to avoid diagnostic and intervention biases (44, 45). Muscle strength is a crucial predictor of functional decline and often deteriorates earlier than muscle mass. Therefore, focusing solely on low muscle mass is insufficient to fully prevent sarcopenia, and early signs of functional decline may be overlooked. For instance, patients with normal muscle mass, but LMS, may experience “muscle dysfunction” or “hidden sarcopenia” (46). This condition is more common in the elderly or in patients with certain chronic diseases and is primarily associated with a reduction in fast-twitch muscle fibers, degeneration of neuromuscular junctions, and muscle metabolic disorders (47). Although the overall muscle volume or mass in these patients remains within normal limits, the muscle function and neuromuscular coordination are significantly impaired. This group faces a higher risk of functional decline and falls during daily activities, highlighting the need for early interventions aimed at improving muscle function, rather than simply maintaining muscle mass. Conversely, patients with low muscle mass, but normal or high muscle strength, may benefit from exercise or genetic factors that help to maintain functional performance. Overall, these findings emphasize that screening based solely on muscle mass or strength fails to capture the complete picture of a patient's muscle health.

Therefore, we conducted a predictive performance analysis. The study revealed that, in the ROC curve, the AUC values of the SII for predicting muscle mass and strength were relatively balanced (0.578 and 0.593, respectively). Although the SII is not the most powerful predictive indicator, its stability surpasses that of the dNLR and PIV. Moreover, among all eight inflammatory markers, only the SII demonstrated a relatively efficient and stable predictive ability for both muscle strength and muscle mass simultaneously, without being influenced by other covariates. To strengthen this conclusion, we included data from 554 patients treated at our hospital for external validation. In the external, training, and internal validation sets, the between-group effect sizes of the SII for predicting muscle mass (AUC = 0.699, 0.677, and 0.685, respectively) and muscle strength (AUC = 0.857, 0.849, and 0.840, respectively) were consistent. These findings indicate that SII has strong practical value as a reference marker to predict low muscle mass and strength.

Our study has some limitations. First, although this study attempted to control for confounding factors by adjusting for covariates such as age, sex, BMI, and smoking status, there may still be residual or unmeasured confounding factors, such as dietary habits, undiagnosed chronic diseases, or medication use. Future studies should refine the collection of participant characteristics to assess the impact of these potential confounders comprehensively. Additionally, this study used cross-sectional data, which limits its ability to infer causal relationships. Future research should incorporate time-series analyses to clarify the causal relationships between the dynamic changes in inflammatory markers and muscle health. Second, CBC blood indicators exhibit a certain degree of variability and may be influenced by short-term factors, such as acute stress, infections, diet, or physical activity, which could affect the stability of the results. Although this study mitigated such variability to some extent by using a large sample size and multicenter data, future research should employ dynamic monitoring of blood indices across multiple time points to evaluate the long-term stability of these markers. Finally, an external validation dataset was derived from the patients at a single medical institution. Although the predictive performance of the SII has been validated, the limited heterogeneity of sample sources may restrict the generalizability of the model. Future studies should include population samples from multiple centers and diverse ethnic backgrounds to ensure the applicability of the model across different populations.

## 5 Conclusion

Although some inflammatory markers (e.g., NMLR, NLR, SIRI, and PIV) were significantly associated with muscle mass and strength in specific quartiles, their independent effects disappeared in the multiple regression models and were highly influenced by BMI. In contrast, the SII proved to be a more balanced marker, demonstrating robust predictive ability and strong independence for muscle mass and strength. More importantly, the SII exhibited a consistent predictive performance in both internal and external validations, underscoring its strong generalizability across different populations. This is particularly important to identify cases of “hidden sarcopenia”.

## Data Availability

The datasets presented in this study can be found in online repositories. The names of the repository/repositories and accession number(s) can be found in the article/Supplementary material.
