# Supplementary material for: The association between eight complete blood count-derived inflammatory markers and muscle health
Source: Front Nutr. 2025 Feb 3;12:1498757. doi: 10.3389/fnut.2025.1498757 (PMC11830586; doi:10.3389/fnut.2025.1498757)
Supplement: Supplementary file 1 [file Data_Sheet_1.docx]

**Supplementary Table 1.** The baseline characteristics by quartiles of the muscle mass: NHANES 2011–2018.

|  | All | Q1 (<0.63) | Q2(0.63–0.79) | Q3(0.79–0.96) | Q4 (≥0.96) | P-value |
| --- | --- | --- | --- | --- | --- | --- |
|  |  |  |  |  |  |  |
|  | N =10440 | N =2610 | N =2610 | N =2610 | N =2610 |  |
| Age, years | 39.33 ± 11.50 | 41.97 ± 11.13 | 38.86 ± 11.42 | 39.84 ± 11.48 | 36.65 ± 11.34 | <0.001 |
| Gender (%) |  |  |  |  |  | <0.001 |
| Male | 5137 (49.20%) | 15 (0.57%) | 416 (15.94%) | 2130 (81.61%) | 2576 (98.70%) |  |
| Female | 5303 (50.80%) | 2595 (99.43%) | 2194 (84.06%) | 480 (18.39%) | 34 (1.30%) |  |
| Race/ethnicity, % |  |  |  |  |  | <0.001 |
| Mexican American | 1569 (15.03%) | 633 (24.25%) | 305 (11.69%) | 445 (17.05%) | 186 (7.13%) |  |
| Non-Hispanic White | 3072 (29.43%) | 863 (33.07%) | 745 (28.54%) | 821 (31.46%) | 643 (24.64%) |  |
| Non-Hispanic Black | 3629 (34.76%) | 784 (30.04%) | 929 (35.59%) | 944 (36.17%) | 972 (37.24%) |  |
| Other Race | 2170 (20.79%) | 330 (12.64%) | 631 (24.18%) | 400 (15.33%) | 809 (31.00%) |  |
| Education level, % |  |  |  |  |  | <0.001 |
| Less Than 9th Grade | 649 (6.22%) | 249 (9.54%) | 128 (4.91%) | 188 (7.20%) | 84 (3.22%) |  |
| 9-11th Grade (Includes 12th grade with no diploma) | 1242 (11.90%) | 354 (13.57%) | 247 (9.47%) | 324 (12.41%) | 317 (12.15%) |  |
| High School Grad/GED or Equivalent | 2280 (21.84%) | 587 (22.50%) | 507 (19.43%) | 574 (21.99%) | 612 (23.45%) |  |
| Some college or AA degree | 3426 (32.82%) | 851 (32.62%) | 945 (36.22%) | 795 (30.46%) | 835 (31.99%) |  |
| College Graduate or above | 2841 (27.22%) | 568 (21.77%) | 782 (29.97%) | 729 (27.93%) | 762 (29.20%) |  |
| Marital (%) |  |  |  |  |  | <0.001 |
| Married | 5099 (48.85%) | 1365 (52.30%) | 1188 (45.53%) | 1372 (52.57%) | 1174 (44.98%) |  |
| Widowed | 144 (1.38%) | 67 (2.57%) | 46 (1.76%) | 20 (0.77%) | 11 (0.42%) |  |
| Divorced | 960 (9.20%) | 291 (11.15%) | 297 (11.38%) | 203 (7.78%) | 169 (6.48%) |  |
| Separated | 370 (3.54%) | 136 (5.21%) | 98 (3.76%) | 86 (3.30%) | 50 (1.92%) |  |
| Never married | 2704 (25.90%) | 463 (17.74%) | 715 (27.41%) | 639 (24.48%) | 887 (33.98%) |  |
| Living with partner | 1162 (11.13%) | 288 (11.03%) | 265 (10.16%) | 290 (11.11%) | 319 (12.22%) |  |
| BMI, kg/m2 | 28.86 ± 6.81 | 31.99 ± 7.66 | 28.52 ± 7.08 | 28.70 ± 6.12 | 26.21 ± 4.73 | <0.001 |
| Diabetes (%) |  |  |  |  |  | <0.001 |
| Yes | 773 (7.40%) | 268 (10.27%) | 179 (6.86%) | 214 (8.20%) | 112 (4.29%) |  |
| No | 9473 (90.74%) | 2275 (87.16%) | 2386 (91.42%) | 2342 (89.73%) | 2470 (94.64%) |  |
| Edge | 194 (1.86%) | 67 (2.57%) | 45 (1.72%) | 54 (2.07%) | 28 (1.07%) |  |
| Moderate recreational activities,n (%) |  |  |  |  |  | <0.001 |
| Inactive | 4661 (44.65%) | 1036 (39.69%) | 1193 (45.71%) | 1169 (44.79%) | 1263 (48.41%) |  |
| Active | 5778 (55.35%) | 1574 (60.31%) | 1417 (54.29%) | 1441 (55.21%) | 1346 (51.59%) |  |
| Smoking |  |  |  |  |  | <0.001 |
| Yes | 2335 (57.30%) | 455 (57.45%) | 505 (55.80%) | 632 (52.62%) | 743 (63.13%) |  |
| No | 1740 (42.70%) | 337 (42.55%) | 400 (44.20%) | 569 (47.38%) | 434 (36.87%) |  |
| PIR | 2.52 ± 1.66 | 2.28 ± 1.61 | 2.57 ± 1.65 | 2.56 ± 1.66 | 2.66 ± 1.68 | <0.001 |
| CBC count, 10^3^/µL |  |  |  |  |  |  |
| White blood cell | 7.28 ± 2.16 | 7.74 ± 2.23 | 7.30 ± 2.19 | 7.30 ± 2.12 | 6.80 ± 1.99 | <0.001 |
| Lymphocyte | 2.24 ± 0.71 | 2.36 ± 0.72 | 2.26 ± 0.72 | 2.22 ± 0.74 | 2.12 ± 0.63 | <0.001 |
| Monocyte | 0.55 ± 0.19 | 0.54 ± 0.18 | 0.53 ± 0.19 | 0.58 ± 0.19 | 0.56 ± 0.19 | <0.001 |
| Neutrophils | 4.25 ± 1.70 | 4.60 ± 1.74 | 4.27 ± 1.73 | 4.25 ± 1.66 | 3.88 ± 1.61 | <0.001 |
| Platelet | 244.31 ± 59.80 | 267.19 ± 65.63 | 251.62 ± 59.26 | 233.95 ± 53.68 | 224.49 ± 50.32 | <0.001 |
| CBC Inflammation index,10 ^3^ /μL |  |  |  |  |  |  |
| MLR | 0.26 ± 0.10 | 0.24 ± 0.09 | 0.25 ± 0.11 | 0.28 ± 0.11 | 0.28 ± 0.11 | <0.001 |
| NMLR | 2.29 ± 1.06 | 2.31 ± 0.96 | 2.28 ± 1.14 | 2.33 ± 1.04 | 2.24 ± 1.10 | 0.017 |
| NLR | 2.03 ± 1.00 | 2.07 ± 0.91 | 2.03 ± 1.08 | 2.05 ± 0.96 | 1.96 ± 1.04 | <0.001 |
| PLR | 117.86 ± 42.82 | 121.84 ± 42.70 | 120.94 ± 45.61 | 114.19 ± 41.23 | 114.48 ± 41.04 | <0.001 |
| dNLR | 0.83 ± 0.05 | 0.85 ± 0.05 | 0.84 ± 0.05 | 0.83 ± 0.05 | 0.82 ± 0.06 | <0.001 |
| SII | 498.67 ± 289.83 | 555.21 ± 289.52 | 512.50 ± 309.87 | 483.37 ± 269.14 | 443.59 ± 277.78 | <0.001 |
| SIRI | 1.15 ± 0.81 | 1.14 ± 0.73 | 1.11 ± 0.81 | 1.22 ± 0.83 | 1.13 ± 0.84 | <0.001 |
| PIV | 287.23 ± 234.51 | 312.00 ± 238.96 | 286.34 ± 240.89 | 290.62 ± 227.54 | 259.96 ± 227.55 | <0.001 |

**Supplementary Table 2.** The baseline characteristics by muscle strength: NHANES 2011–2014.

|  | All | Q1(<0.97) | Q2(0.97-1.27) | Q3(1.27-1.61) | Q4(＞1.61) | P-value |
| --- | --- | --- | --- | --- | --- | --- |
|  | N=5384 | N=1314 | N=1352 | N=1352 | N=1366 |  |
| Age, years | 38.95 ± 11.52 | 41.58 ± 11.41 | 39.01 ± 11.46 | 38.88 ± 11.43 | 36.42 ± 11.23 | <0.001 |
| Gender (%) |  |  |  |  |  | <0.001 |
| Male | 2741 (50.91%) | 111 (8.45%) | 410 (30.33%) | 902 (66.72%) | 1318 (96.49%) |  |
| Female | 2643 (49.09%) | 1203 (91.55%) | 942 (69.67%) | 450 (33.28%) | 48 (3.51%) |  |
| Race/ethnicity, % |  |  |  |  |  | <0.001 |
| Mexican American | 690 (12.82%) | 237 (18.04%) | 167 (12.35%) | 162 (11.98%) | 124 (9.08%) |  |
| Non-Hispanic White | 1411 (26.21%) | 307 (23.36%) | 376 (27.81%) | 393 (29.07%) | 335 (24.52%) |  |
| Non-Hispanic Black | 2120 (39.38%) | 458 (34.86%) | 509 (37.65%) | 551 (40.75%) | 602 (44.07%) |  |
| Other Race | 1163 (21.60%) | 312 (23.74%) | 300 (22.19%) | 246 (18.20%) | 305 (22.33%) |  |
| Education level, % |  |  |  |  |  | <0.001 |
| Less Than 9th Grade | 264 (4.90%) | 83 (6.32%) | 64 (4.73%) | 61 (4.51%) | 56 (4.10%) |  |
| 9-11^th^ Grade (Includes 12th grade with no diploma) | 682 (12.67%) | 192 (14.62%) | 161 (11.91%) | 141 (10.43%) | 188 (13.76%) |  |
| High School Grad/GED or Equivalent | 1143 (21.23%) | 301 (22.92%) | 253 (18.71%) | 266 (19.67%) | 323 (23.65%) |  |
| Some college or AA degree | 1793 (33.31%) | 476 (36.25%) | 465 (34.39%) | 436 (32.25%) | 416 (30.45%) |  |
| College Graduate or above | 1501 (27.88%) | 261 (19.88%) | 409 (30.25%) | 448 (33.14%) | 383 (28.04%) |  |
| Marital (%) |  |  |  |  |  | <0.001 |
| Married | 2593 (48.16%) | 607 (46.19%) | 651 (48.15%) | 698 (51.63%) | 637 (46.63%) |  |
| Widowed | 69 (1.28%) | 32 (2.44%) | 17 (1.26%) | 9 (0.67%) | 11 (0.81%) |  |
| Divorced | 524 (9.73%) | 170 (12.94%) | 135 (9.99%) | 120 (8.88%) | 99 (7.25%) |  |
| Separated | 188 (3.49%) | 63 (4.79%) | 51 (3.77%) | 40 (2.96%) | 34 (2.49%) |  |
| Never married | 1468 (27.27%) | 319 (24.28%) | 367 (27.14%) | 359 (26.55%) | 423 (30.97%) |  |
| Living with partner | 542 (10.07%) | 123 (9.36%) | 131 (9.69%) | 126 (9.32%) | 162 (11.86%) |  |
| BMI, kg/m2 | 28.63 ± 6.76 | 34.48 ± 7.56 | 28.35 ± 5.83 | 26.99 ± 5.24 | 24.90 ± 3.81 | <0.001 |
| Diabetes (%) |  |  |  |  |  | <0.001 |
| Yes | 377 (7.00%) | 166 (12.63%) | 101 (7.47%) | 71 (5.25%) | 39 (2.86%) |  |
| No | 4909 (91.18%) | 1107 (84.25%) | 1226 (90.68%) | 1260 (93.20%) | 1316 (96.34%) |  |
| Edge | 98 (1.82%) | 41 (3.12%) | 25 (1.85%) | 21 (1.55%) | 11 (0.81%) |  |
| Moderate recreational activities,n (%) |  |  |  |  |  | <0.001 |
| Inactive | 2382 (44.24%) | 517 (39.35%) | 608 (44.97%) | 628 (46.45%) | 629 (46.05%) |  |
| Active | 3002 (55.76%) | 797 (60.65%) | 744 (55.03%) | 724 (53.55%) | 737 (53.95%) |  |
| Smoking |  |  |  |  |  | <0.001 |
| Yes | 1276 (58.64%) | 271 (57.78%) | 260 (53.94%) | 301 (54.83%) | 444 (65.68%) |  |
| No | 900 (41.36%) | 198 (42.22%) | 222 (46.06%) | 248 (45.17%) | 232 (34.32%) |  |
| PIR | 2.52 ± 1.69 | 2.23 ± 1.60 | 2.55 ± 1.70 | 2.70 ± 1.72 | 2.59 ± 1.70 | <0.001 |
| CBC count, 103/µL |  |  |  |  |  |  |
| White blood cell | 7.21 ± 2.14 | 7.77 ± 2.34 | 7.24 ± 2.11 | 6.98 ± 1.97 | 6.87 ± 2.02 | <0.001 |
| Lymphocyte | 2.19 ± 0.68 | 2.33 ± 0.72 | 2.19 ± 0.69 | 2.13 ± 0.63 | 2.10 ± 0.64 | <0.001 |
| Monocyte | 0.54 ± 0.19 | 0.54 ± 0.18 | 0.53 ± 0.19 | 0.54 ± 0.19 | 0.55 ± 0.18 | 0.058 |
| Neutrophils | 4.25 ± 1.70 | 4.67 ± 1.87 | 4.28 ± 1.67 | 4.07 ± 1.56 | 3.98 ± 1.62 | <0.001 |
| Platelet | 241.49 ± 58.90 | 263.99 ± 64.09 | 245.80 ± 56.92 | 230.36 ± 54.72 | 226.62 ± 52.07 | <0.001 |
| CBC Inflammation index,10^3^ /μL |  |  |  |  |  |  |
| MLR | 0.26 ± 0.11 | 0.24 ± 0.09 | 0.26 ± 0.11 | 0.27 ± 0.11 | 0.28 ± 0.11 | <0.001 |
| NMLR | 2.33 ± 1.03 | 2.36 ± 0.96 | 2.35 ± 1.01 | 2.32 ± 1.07 | 2.31 ± 1.09 | 0.585 |
| NLR | 2.07 ± 0.97 | 2.12 ± 0.92 | 2.09 ± 0.94 | 2.05 ± 1.01 | 2.03 ± 1.02 | 0.109 |
| PLR | 119.25 ± 42.73 | 121.98 ± 42.11 | 121.45 ± 43.04 | 116.89 ± 44.05 | 116.79 ± 41.44 | <0.001 |
| dNLR | 0.84 ± 0.05 | 0.85 ± 0.05 | 0.84 ± 0.05 | 0.83 ± 0.05 | 0.82 ± 0.06 | <0.001 |
| SII | 504.08 ± 288.69 | 561.78 ± 289.89 | 515.05 ± 266.69 | 475.21 ± 303.98 | 466.29 ± 283.54 | <0.001 |
| SIRI | 1.15 ± 0.77 | 1.16 ± 0.72 | 1.14 ± 0.76 | 1.14 ± 0.78 | 1.15 ± 0.82 | 0.895 |
| PIV | 282.81 ± 225.17 | 311.35 ± 225.23 | 284.14 ± 208.44 | 268.04 ± 237.43 | 268.64 ± 226.16 | <0.001 |

**Supplementary Table 3.** Weighted logistic and linear regression analysis models for the association between inflammatory biomarkers and muscle mass.

| Exposure | Continuous or categories | Model 1 |  | Model 2 |  | Model 3 |  |
| --- | --- | --- | --- | --- | --- | --- | --- |
|  |  | (β, 95% CI) | P-value | (β, 95% CI) | P-value | (β, 95% CI) | P-value |
| MLR | MLR as a continuous variable | 0.29 (0.26, 0.33) | <0.001 | 0.02 (0.00, 0.05) | 0.028 | 0.00 (-4.70, 4.70) | 0.999 |
|  | Q1 | Ref |  | Ref |  | Ref |  |
|  | Q2 | 0.02 (0.01, 0.04) | <0.001 | 0.00 (-0.01, 0.01) | 0.761 | 0.00 (-0.02, 0.01) | 0.757 |
|  | Q3 | 0.05 (0.04, 0.06) | <0.001 | 0.01 (0.00, 0.01) | 0.048 | 0.00 (-0.02, 0.02) | 0.995 |
|  | Q4 | 0.09 (0.08, 0.10) | <0.001 | 0.01 (0.00, 0.01) | 0.042 | 0.00 (-0.02, 0.01) | 0.909 |
| NMLR | NMLR as a continuous variable | -0.00 (-0.01, -0.00) | 0.033 | -0.00 (-0.00, -0.00) | 0.010 | -0.00 (-0.01, 0.00) | 0.123 |
|  | Q1 | Ref |  | Ref |  | Ref |  |
|  | Q2 | -0.03 (-0.04, -0.02) | <0.001 | -0.01 (-0.02, -0.00) | 0.005 | 0.01 (-0.03, 0.00) | 0.117 |
|  | Q3 | -0.02 (-0.03, -0.01) | <0.001 | -0.01 (-0.02, -0.01) | <0.001 | 0.00 (-0.02, 0.01) | 0.554 |
|  | Q4 | -0.03 (-0.04, -0.02) | <0.001 | -0.02 (-0.02, -0.01) | <0.001 | -0.02 (-0.04, -0.01) | 0.002 |
| NLR | NLR as a continuous variable | -0.01 (-0.01, -0.00) | 0.001 | -0.00 (-0.01, -0.00) | 0.003 | -0.00 (-0.01, 0.00) | 0.102 |
|  | Q1 | Ref |  | Ref |  | Ref |  |
|  | Q2 | -0.03 (-0.05, -0.02) | <0.001 | -0.01 (-0.02, -0.00) | 0.004 | -0.01 (-0.03, 0.00) | 0.096 |
|  | Q3 | -0.03 (-0.04, -0.02) | <0.001 | -0.01 (-0.02, -0.01) | <0.001 | -0.01 (-0.02, 0.01) | 0.404 |
|  | Q4 | -0.04 (-0.05, -0.03) | <0.001 | -0.02 (-0.02, -0.01) | <0.001 | -0.02 (-0.04, -0.01) | 0.002 |
|  | Exposure | Non-adjusted |  | Adjust I |  | Adjust II |  |
| dNLR | dNLR as a continuous variable | -0.81 (-0.88, -0.74) | <0.001 | -0.16 (-0.20, -0.12) | <0.001 | -0.16 (-0.26, -0.07) | 0.001 |
|  | Q1 | Ref |  | Ref |  | Ref |  |
|  | Q2 | -0.04 (-0.05, -0.03) | <0.001 | -0.00 (-0.01, 0.00) | 0.306 | -0.00 (-0.02, 0.01) | 0.684 |
|  | Q3 | -0.08 (-0.09, -0.07) | <0.001 | -0.02 (-0.02, -0.01) | <0.001 | -0.01 (-0.03, 0.00) | 0.120 |
|  | Q4 | -0.11 (-0.12, -0.10) | <0.001 | -0.02 (-0.03, -0.01) | <0.001 | -0.02 (-0.04, -0.01) | 0.005 |
| SII | SII as a continuous variable | -0.00 (-0.00, -0.00) | <0.001 | -0.00 (-0.00, -0.00) | <0.001 | -0.00 (-0.00, -0.00) | 0.002 |
|  | Q1 | Ref |  | Ref |  | Ref |  |
|  | Q2 | -0.04 (-0.05, -0.03) | <0.001 | -0.02 (-0.02, -0.01) | <0.001 | -0.02 (-0.03, -0.00) | 0.027 |
|  | Q3 | -0.07 (-0.08, -0.06) | <0.001 | -0.03 (-0.03, -0.02) | <0.001 | -0.02 (-0.03, -0.00) | 0.012 |
|  | Q4 | -0.10 (-0.11, -0.09) | <0.001 | -0.03 (-0.04, -0.03) | <0.001 | -0.03 (-0.04, -0.02) | <0.001 |
| SIRI | SIRI as a continuous variable | 0.00 (-0.00, 0.01) | 0.645 | -0.01 (-0.01, -0.01) | <0.001 | -0.01 (-0.02, -0.00) | 0.001 |
|  | Q1 | Ref |  | Ref |  | Ref |  |
|  | Q2 | -0.02 (-0.03, -0.00) | 0.008 | -0.01 (-0.02, -0.00) | 0.001 | -0.01 (-0.02, 0.01) | 0.292 |
|  | Q3 | -0.02 (-0.03, -0.01) | <0.001 | -0.02 (-0.03, -0.01) | <0.001 | -0.01 (-0.03, 0.00) | 0.095 |
|  | Q4 | -0.01 (-0.02, 0.00) | 0.055 | -0.03 (-0.03, -0.02) | <0.001 | -0.03 (-0.04, -0.01) | <0.001 |
| PLR | PLR as a continuous variable | -0.00 (-0.00, -0.00) | <0.001 | 0.00 (-0.00, 0.00) | 0.116 | 0.01 (-0.00, 0.02) | 0.170 |
|  | Q1 | Ref |  | Ref |  | Ref |  |
|  | Q2 | -0.02 (-0.03, -0.01) | 0.003 | 0.00 (-0.00, 0.01) | 0.665 | 0.00 (-0.01, 0.02) | 0.606 |
|  | Q3 | -0.02 (-0.04, -0.01) | <0.001 | 0.00 (-0.00, 0.01) | 0.452 | 0.00 (-0.02, 0.01) | 0.777 |
|  | Q4 | -0.04 (-0.05, -0.03) | <0.001 | 0.00 (-0.00, 0.01) | 0.392 | 0.01 (-0.00, 0.03) | 0.074 |
| PIV | PIV as a continuous variable | -0.00 (-0.00, -0.00) | <0.001 | -0.00 (-0.00, -0.00) | <0.001 | -0.004 (-0.01, -0.00) | <0.001 |
|  | Q1 | Ref |  | Ref |  | Ref |  |
|  | Q2 | -0.03 (-0.04, -0.02) | <0.001 | -0.02 (-0.03, -0.01) | <0.001 | -0.01 (-0.03, 0.00) | 0.122 |
|  | Q3 | -0.05 (-0.06, -0.04) | <0.001 | -0.03 (-0.03, -0.02) | <0.001 | -0.02 (-0.04, -0.01) | 0.003 |
|  | Q4 | -0.06 (-0.07, -0.05) | <0.001 | -0.04 (-0.05, -0.03) | <0.001 | -0.03 (-0.05, -0.02) | <0.001 |

Model 1: No adjustments were made. Model 2: Adjusted for age, sex, race, poverty-income ratio (PIR), and education. Model 3: Based on Model 2, additional adjustments were made for smoking status, diabetic status, BMI, and moderate recreational activities.

**Supplementary Table 4.** Weighted logistic and linear regression analysis models for the association between inflammatory biomarkers and muscle strength.

| Exposure | Continuous or categories | Model 1 |  | Model 2 |  | Model 3 |  |
| --- | --- | --- | --- | --- | --- | --- | --- |
|  |  | (β, 95% CI) | P-value | (β, 95% CI) | P-value | (β, 95% CI) | P-value |
| MLR | MLR as a continuous variable | 0.55 (0.43, 0.66) | <0.001 | 0.05 (-0.04, 0.13) | 0.256 | -0.17 (-0.32, -0.01) | 0.337 |
|  | Q1 | Ref |  | Ref |  | Ref |  |
|  | Q2 | 0.04 (0.01, 0.07) | 0.020 | 0.00 (-0.02, 0.03) | 0.902 | -0.01 (-0.05, 0.03) | 0.642 |
|  | Q3 | 0.12 (0.08, 0.15) | <0.001 | 0.01 (-0.02, 0.04) | 0.501 | -0.02 (-0.07, 0.03) | 0.408 |
|  | Q4 | 0.17 (0.13, 0.20) | <0.001 | 0.02 (-0.01, 0.04) | 0.025 | -0.06 (-0.11, -0.01) | 0.013 |
| NMLR | NMLR as a continuous variable | -0.01 (-0.02, 0.00) | 0.245 | -0.01 (-0.02, -0.00) | 0.013 | -0.04 (-0.06, -0.02) | <0.001 |
|  | Q1 | Ref |  | Ref |  | Ref |  |
|  | Q2 | -0.04 (-0.07, -0.01) | 0.018 | -0.02 (-0.05, 0.00) | 0.075 | -0.07 (-0.11, -0.02) | 0.003 |
|  | Q3 | -0.04 (-0.07, -0.00) | 0.038 | -0.04 (-0.06, -0.01) | 0.005 | -0.09 (-0.13, -0.04) | <0.001 |
|  | Q4 | -0.05 (-0.09, -0.02) | 0.002 | -0.06 (-0.08, -0.03) | <0.001 | -0.09 (-0.13, -0.04) | <0.001 |
| NLR | NLR as a continuous variable | -0.01 (-0.03, -0.00) | 0.024 | -0.01 (-0.02, -0.00) | 0.006 | -0.01 (-0.03, 0.01) | 0.193 |
|  | Q1 | Ref |  | Ref |  | Ref |  |
|  | Q2 | -0.04 (-0.07, -0.00) | 0.033 | -0.02 (-0.04, 0.01) | 0.219 | -0.06 (-0.11, -0.02) | 0.006 |
|  | Q3 | -0.04 (-0.08, -0.01) | 0.011 | -0.03 (-0.06, -0.01) | 0.007 | -0.08 (-0.13, -0.04) | <0.001 |
|  | Q4 | -0.07 (-0.10, -0.04) | <0.001 | -0.06 (-0.08, -0.03) | <0.001 | -0.09 (-0.13, -0.04) | <0.001 |
| dNLR | dNLR as a continuous variable | -1.34 (-1.55, -1.12) | <0.001 | -0.33 (-0.49, -0.16) | <0.001 | -0.04 (-0.33, 0.26) | 0.892 |
|  | Q1 | Ref |  | Ref |  | Ref |  |
|  | Q2 | -0.05 (-0.09, -0.01) | 0.006 | 0.00 (-0.02, 0.03) | 0.773 | 0.00 (-0.04, 0.05) | 0.889 |
|  | Q3 | -0.12 (-0.16, -0.09) | <0.001 | -0.03 (-0.05, -0.00) | 0.036 | -0.04 (-0.08, 0.00) | 0.059 |
|  | Q4 | -0.20 (-0.23, -0.16) | <0.001 | -0.05 (-0.08, -0.03) | <0.001 | -0.01 (-0.05, 0.03) | 0.685 |
| SII | SII as a continuous variable | -0.00 (-0.00, -0.00) | <0.001 | -0.00 (-0.00, -0.00) | <0.001 | -0.01 (-0.02, -0.00) | 0.001 |
|  | Q1 | Ref |  | Ref |  | Ref |  |
|  | Q2 | -0.07 (-0.10, -0.04) | <0.001 | -0.03 (-0.05, -0.01) | 0.018 | -0.02 (-0.07, 0.02) | 0.293 |
|  | Q3 | -0.11 (-0.14, -0.07) | <0.001 | -0.06 (-0.09, -0.04) | <0.001 | -0.07 (-0.11, -0.03) | 0.001 |
|  | Q4 | -0.20 (-0.23, -0.17) | <0.001 | -0.10 (-0.12, -0.07) | <0.001 | -0.07 (-0.11, -0.03) | 0.002 |
| SIRI | SIRI as a continuous variable | 0.00 (-0.01, 0.02) | 0.835 | -0.03 (-0.04, -0.02) | <0.001 | -0.02 (-0.04, -0.00) | 0.016 |
|  | Q1 | Ref |  | Ref |  | Ref |  |
|  | Q2 | -0.01 (-0.04, 0.02) | 0.552 | -0.03 (-0.05, -0.00) | 0.030 | -0.01 (-0.06, 0.03) | 0.579 |
|  | Q3 | -0.01 (-0.05, 0.02) | 0.394 | -0.04 (-0.07, -0.02) | 0.001 | -0.04 (-0.08, 0.01) | 0.110 |
|  | Q4 | -0.03 (-0.06, 0.01) | 0.1053 | -0.09 (-0.12, -0.07) | <0.001 | -0.07 (-0.12, -0.03) | 0.001 |
| PLR | PLR as a continuous variable | -0.00 (-0.00, -0.00) | <0.001 | 0.00 (0.00, 0.00) | 0.010 | -0.01 (-0.06, 0.05) | 0.984 |
|  | Q1 | Ref |  | Ref |  | Ref |  |
|  | Q2 | -0.02 (-0.06, 0.01) | 0.168 | 0.00 (-0.02, 0.03) | 0.832 | -0.03 (-0.07, 0.01) | 0.139 |
|  | Q3 | -0.04 (-0.07, -0.01) | 0.021 | 0.01 (-0.01, 0.03) | 0.408 | -0.02 (-0.06, 0.03) | 0.519 |
|  | Q4 | -0.07 (-0.10, -0.04) | <0.001 | 0.02 (-0.01, 0.04) | 0.148 | -0.03 (-0.09, 0.02) | 0.228 |
| PIV | PIV as continuous variable | -0.00 (-0.00, -0.00) | <0.001 | -0.00 (-0.00, -0.00) | <0.001 | -0.01 (-0.01, -0.00) | 0.014 |
|  | Q1 | Ref |  | Ref |  | Ref |  |
|  | Q2 | -0.02 (-0.05, 0.01) | 0.248 | -0.03 (-0.05, -0.00) | 0.038 | -0.02 (-0.06, 0.02) | 0.401 |
|  | Q3 | -0.05 (-0.08, -0.02) | 0.003 | -0.05 (-0.07, -0.02) | <0.001 | -0.01 (-0.06, 0.03) | 0.547 |
|  | Q4 | -0.12 (-0.15, -0.08) | <0.001 | -0.11 (-0.13, -0.08) | <0.001 | -0.07 (-0.11, -0.03) | 0.001 |

Model 1: No adjustments were made. Model 2: Adjusted for age, sex, race, poverty-income ratio (PIR), and education. Model 3: Based on Model 2, additional adjustments were made for smoking status, diabetic status, and moderate recreational activities.

**Supplementary Table 5.** Analysis of threshold effects.

|  |  | Muscle mass |  | Muscle strength |  |
| --- | --- | --- | --- | --- | --- |
|  |  | β (95% CI) | P-value | β (95% CI) | P-value |
| MLR |  |  |  |  |  |
|  | Fitting by linear regression model | 0.00 (-0.05, 0.05) | 0.999 | -0.17 (-0.32, -0.01) | 0.337 |
|  | Fitting by two-piecewise linear regression model |  |  |  |  |
|  | Inflection point | 0.15 |  | 0.38 |  |
|  | < K | 0.46 (-0.15, 1.07) | 0.147 | -0.33 (-0.56, -0.09) | 0.007 |
|  | > K | -0.01 (-0.06, 0.04) | 0.652 | 0.06 (-0.24, 0.35) | 0.715 |
|  | Log-likelihood ratio | 0.14 |  | 0.08 |  |
| NMLR |  |  |  |  |  |
|  | Fitting by linear regression model | -0.00 (-0.01, 0.00) | 0.123 | -0.04 (-0.06, -0.02) | <0.001 |
|  | Fitting by two-piecewise linear regression model |  |  |  |  |
|  | Inflection point | 2.88 |  | 2.68 |  |
|  | < K | -0.01 (-0.02, -0.00) | 0.007 | -0.08 (-0.11, -0.05) | <0.001 |
|  | > K | 0.00 (-0.00, 0.01) | 0.417 | 0.00 (-0.03, 0.04) | 0.864 |
|  | Log-likelihood ratio | 0.023 |  | <0.001 |  |
| NLR |  |  |  |  |  |
|  | Fitting by linear regression model | -0.00 (-0.01, 0.00) | 0.102 | -0.01 (-0.03, 0.01) | 0.193 |
|  | Fitting by two-piecewise linear regression model |  |  |  |  |
|  | Inflection point | 2.63 |  | 2.37 |  |
|  | < K | -0.02 (-0.02, -0.01) | <0.001 | -0.09(-0.12, -0.05) | <0.001 |
|  | > K | 0.00 (-0.00, 0.01) | 0.334 | -0.00 (-0.04, 0.03) | 0.905 |
|  | Log-likelihood ratio | 0.012 |  | <0.001 |  |
| dNLR |  |  |  |  |  |
|  | Fitting by linear regression model | -0.16 (-0.26, -0.07) | 0.001 | -0.04 (-0.33, 0.26) | 0.892 |
|  | Fitting by two-piecewise linear regression model |  |  |  |  |
|  | Inflection point | 0.77 |  | 0.86 |  |
|  | < K | -0.24 (-0.51, 0.02) | 0.075 | -0.16 (-0.53, 0.20) | 0.387 |
|  | > K | -0.14 (-0.26, -0.01) | 0.037 | 0.63 (-0.23, 1.49) | 0.151 |
|  | Log-likelihood ratio | 0.534 |  | 0.140 |  |
| SII |  |  |  |  |  |
|  | Fitting by linear regression model | -0.00 (-0.00, -0.00) | 0.002 | -0.01 (-0.02, -0.00) | 0.001 |
|  | Fitting by two-piecewise linear regression model |  |  |  |  |
|  | Inflection point | 608.21 |  | 100.21 |  |
|  | < K | -0.00 (-0.01, -0.00) | <0.001 | -0.01 (-0.02, -0.01) | 0.001 |
|  | > K | 0.00 (-0.00, 0.01) | 0.263 | 0.02 (-0.02, 0.06) | 0.278 |
|  | Log-likelihood ratio | 0.002 |  | <0.001 |  |
| SIRI |  |  |  |  |  |
|  | Fitting by linear regression model | -0.01 (-0.02, -0.01) | <0.001 | -0.02 (-0.04, -0.00) | 0.016 |
|  | Fitting by two-piecewise linear regression model |  |  |  |  |
|  | Inflection point | 1.77 |  | 1.50 |  |
|  | < K | -0.03 (-0.04, -0.01) | <0.001 | -0.09 (-0.14, -0.05) | <0.001 |
|  | > K | 0.01 (-0.01, 0.03) | 0.504 | 0.02 (-0.01, 0.04) | 0.291 |
|  | Log-likelihood ratio | 0.017 |  | <0.001 |  |
| PIV |  |  |  |  |  |
|  | Fitting by linear regression model | -0.00 (-0.01, -0.00) | <0.001 | -0.01 (-0.01, -0.00) | 0.014 |
|  | Fitting by two-piecewise linear regression model |  |  |  |  |
|  | Inflection point | 355.40 |  | 452.80 |  |
|  | < K | -0.17 (-0.28, -0.06) | 0.003 | -0.03 (-0.04, -0.01) | <0.001 |
|  | > K | -0.00 (-0.01, -0.00) | <0.001 | 0.01 (-0.01, 0.02) | 0.342 |
|  | Log-likelihood ratio | 0.004 |  | 0.002 |  |
| PLR |  |  |  |  |  |
|  | Fitting by linear regression model | 0.01 (-0.00, 0.02) | 0.170 | -0.01 (-0.06, 0.05) | 0.775 |
|  | Fitting by two-piecewise linear regression model |  |  |  |  |
|  | Inflection point | 74.43 |  | 98.60 |  |
|  | < K | 0.11 (0.01, 0.21) | 0.026 | -0.10 (-0.24, 0.05) | 0.181 |
|  | > K | 0.00 (-0.01, 0.02) | 0.674 | 0.02 (-0.04, 0.06) | 0.564 |
|  | Log-likelihood ratio | 0.036 |  | 0.154 |  |

Threshold Effect Analyses:All models were adjusted for age, sex, race, education level, poverty-income ratio (PIR), diabetes status, marital status, smoking habits, and moderate recreational activity.

**Supplementary Table 6.** Comparison of AUC values for the eight inflammatory markers in sarcopenia and LMS, respectively.

| Test | AUC | 95%CI low | 95%CI upp | Best threshold | Specificity | Sensitivity | P for different in AUC |
| --- | --- | --- | --- | --- | --- | --- | --- |
| Low muscle mass |  |  |  |  |  |  |  |
| NMLR | 0.553 | 0.534 | 0.572 | 2.425 | 0.659 | 0.425 | Ref |
| MLR | 0.507 | 0.487 | 0.526 | 0.215 | 0.642 | 0.387 | 0.010 |
| NLR | 0.559 | 0.540 | 0.578 | 1.685 | 0.418 | 0.673 | <0.001 |
| PLR | 0.514 | 0.495 | 0.534 | 118.870 | 0.421 | 0.604 | 0.023 |
| dNLR | 0.571 | 0.552 | 0.590 | 0.835 | 0.468 | 0.647 | 0.066 |
| SII | 0.578 | 0.559 | 0.598 | 502.825 | 0.624 | 0.517 | <0.001 |
| SIRI | 0.569 | 0.550 | 0.588 | 1.275 | 0.699 | 0.399 | 0.004 |
| PIV | 0.583 | 0.564 | 0.603 | 262.745 | 0.594 | 0.530 | <0.001 |
|  |  |  |  |  |  |  |  |
| Low muscle strength | |  |  |  |  |  |  |
| NMLR | 0.526 | 0.508 | 0.543 | 2.535 | 0.687 | 0.368 | Ref |
| MLR | 0.574 | 0.557 | 0.591 | 0.255 | 0.480 | 0.652 | 0.002 |
| NLR | 0.535 | 0.518 | 0.553 | 2.095 | 0.614 | 0.455 | <0.001 |
| PLR | 0.533 | 0.516 | 0.551 | 123.96 | 0.635 | 0.433 | 0.421 |
| dNLR | 0.596 | 0.579 | 0.614 | 0.855 | 0.641 | 0.511 | <0.001 |
| SII | 0.593 | 0.576 | 0.611 | 490.225 | 0.618 | 0.531 | <0.001 |
| SIRI | 0.519 | 0.501 | 0.537 | 1.135 | 0.626 | 0.414 | 0.216 |
| PIV | 0.566 | 0.548 | 0.584 | 256.660 | 0.608 | 0.505 | <0.001 |

**Supplementary Table 7.** Baseline characteristics according to muscle mass and muscle strength quartiles: 554 patients in external hospitals

|  | Muscle mass | | | |  | Muscle strength | | | |  |
| --- | --- | --- | --- | --- | --- | --- | --- | --- | --- | --- |
|  | Q1(<0.64) | Q2(0.64-0.78) | Q3(0.78-0.96) | Q4(>0.96) | P-value | Q1(<0.96) | Q2(0.96-1.24) | Q3(1.24-1.55) | Q4(>1.55) | P-value |
| All=554 | N=139 | N=138 | N=138 | N=139 |  | N=134 | N=143 | N=138 | N=139 |  |
| Age, years | 42.53 ± 10.69 | 38.49 ± 10.46 | 39.65 ± 10.90 | 37.35 ± 10.80 | <0.001 | 43.01 ± 10.81 | 39.39 ± 10.46 | 37.97 ± 11.09 | 37.76 ± 10.36 | <0.001 |
| Gender (%) |  |  |  |  | <0.001 |  |  |  |  | <0.001 |
| Male | 1 (0.719%) | 15 (10.870%) | 118 (85.507%) | 138 (99.281%) |  | 11 (8.209%) | 39 (27.273%) | 91 (65.942%) | 131 (94.245%) |  |
| Female | 138 (99.281%) | 123 (89.130%) | 20 (14.493%) | 1 (0.719%) |  | 123 (91.791%) | 104 (72.727%) | 47 (34.058%) | 8 (5.755%) |  |
| Education level, % |  |  |  |  | 0.026 |  |  |  |  | 0.002 |
| Below junior high school | 22 (15.827%) | 20 (14.493%) | 29 (21.014%) | 21 (15.108%) |  | 25 (18.657%) | 17 (11.888%) | 28 (20.290%) | 22 (15.827%) |  |
| Middle to high school | 43 (30.935%) | 21 (15.217%) | 30 (21.739%) | 28 (20.144%) |  | 41 (30.597%) | 21 (14.685%) | 23 (16.667%) | 37 (26.619%) |  |
| University or post-secondary | 74 (53.237%) | 97 (70.290%) | 79 (57.246%) | 90 (64.748%) |  | 68 (50.746%) | 105 (73.427%) | 87 (63.043%) | 80 (57.554%) |  |
| Marital (%) |  |  |  |  | 0.009 |  |  |  |  | 0.61 |
| Married | 97 (69.784%) | 107 (77.536%) | 95 (68.841%) | 117 (84.173%) |  | 100 (74.627%) | 102 (71.329%) | 106 (76.812%) | 108 (77.698%) |  |
| Unmarried or divorced | 42 (30.216%) | 31 (22.464%) | 43 (31.159%) | 22 (15.827%) |  | 34 (25.373%) | 41 (28.671%) | 32 (23.188%) | 31 (22.302%) |  |
| Diabetes (%) |  |  |  |  | 0.116 |  |  |  |  | <0.001 |
| Yes | 22 (15.827%) | 13 (9.420%) | 14 (10.145%) | 10 (7.194%) |  | 24 (17.910%) | 21 (14.685%) | 10 (7.246%) | 4 (2.878%) |  |
| No | 117 (84.173%) | 125 (90.580%) | 124 (89.855%) | 129 (92.806%) |  | 110 (82.090%) | 122 (85.315%) | 128 (92.754%) | 135 (97.122%) |  |
| Moderate recreational activities,n (%) |  |  |  |  | 0.034 |  |  |  |  | 0.002 |
| Inactive | 51 (36.691%) | 61 (44.203%) | 59 (42.754%) | 75 (53.957%) |  | 47 (35.075%) | 62 (43.357%) | 57 (41.304%) | 80 (57.554%) |  |
| Active | 88 (63.309%) | 77 (55.797%) | 79 (57.246%) | 64 (46.043%) |  | 87 (64.925%) | 81 (56.643%) | 81 (58.696%) | 59 (42.446%) |  |
| Smoking |  |  |  |  | 0.324 |  |  |  |  | 0.875 |
| Yes | 34 (24.460%) | 43 (31.159%) | 47 (34.058%) | 45 (32.374%) |  | 40 (29.851%) | 41 (28.671%) | 42 (30.435%) | 46 (33.094%) |  |
| No | 105 (75.540%) | 95 (68.841%) | 91 (65.942%) | 94 (67.626%) |  | 94 (70.149%) | 102 (71.329%) | 96 (69.565%) | 93 (66.906%) |  |
| BMI,kg/m2 | 31.66 ± 8.66 | 28.23 ± 6.79 | 28.85 ± 5.77 | 26.67 ± 4.18 | <0.001 | 34.03 ± 8.62 | 28.87 ± 5.62 | 27.32 ± 5.21 | 25.37 ± 3.58 | <0.001 |
| CBC count, 103/µL |  |  |  |  |  |  |  |  |  |  |
| White blood cell | 7.60 ± 2.01 | 6.96 ± 1.89 | 7.45 ± 2.12 | 6.64 ± 1.87 | <0.001 | 7.63 ± 2.15 | 7.25 ± 1.93 | 6.91 ± 1.91 | 6.865 ± 1.96 | 0.005 |
| Lymphocyte | 2.28 ± 0.71 | 2.17± 0.69 | 2.24 ± 0.70 | 2.08 ± 0.60 | 0.081 | 2.29 ± 0.79 | 2.22 ± 0.62 | 2.12 ± 0.66 | 2.14 ± 0.63 | 0.133 |
| Monocyte | 0.52 ± 0.16 | 0.51 ± 0.17 | 0.58 ± 0.18 | 0.55 ± 0.18 | 0.009 | 0.53 ± 0.17 | 0.54 ± 0.17 | 0.54 ± 0.19 | 0.56 ± 0.18 | 0.620 |
| Neutrophils | 4.55 ± 1.64 | 4.06 ± 1.50 | 4.39 ± 1.72 | 3.73 ± 1.39 | <0.001 | 4.56 ± 1.74 | 4.245 ± 1.57 | 4.04 ± 1.58 | 3.89 ± 1.43 | 0.003 |
| Platelet | 262.58 ± 61.29 | 251.85 ± 55.29 | 237.32 ± 54.90 | 219.72 ± 44.73 | <0.001 | 261.96 ± 61.92 | 252.67 ± 55.52 | 232.96 ± 55.57 | 224.18 ± 44.61 | <0.001 |
| MLR | 0.24 ± 0.09 | 0.25 ± 0.10 | 0.28 ± 0.10 | 0.28 ± 0.10 | 0.011 | 0.25 ± 0.10 | 0.26 ± 0.10 | 0.27 ± 0.10 | 0.27 ± 0.10 | 0.129 |
| NMLR | 2.38 ± 0.94 | 2.26 ± 0.97 | 2.38 ± 0.99 | 2.16 ± 0.81 | 0.163 | 2.41 ± 1.05 | 2.28 ± 0.86 | 2.32 ± 1.03 | 2.180 ± 0.77 | 0.223 |
| NLR | 2.13 ± 0.90 | 2.01 ± 0.91 | 2.10 ± 0.93 | 1.88 ± 0.74 | 0.088 | 2.16 ± 0.99 | 2.02 ± 0.80 | 2.05 ± 0.97 | 1.90 ± 0.70 | 0.118 |
| PLR | 123.22 ± 39.63 | 127.52 ± 49.16 | 114.53 ± 39.15 | 112.48 ± 34.16 | 0.006 | 124.38 ± 42.92 | 122.58 ± 43.95 | 118.80 ± 41.97 | 112.06 ± 34.79 | 0.064 |
| DNLR | 0.84 ± 0.05 | 0.83 ± 0.05 | 0.82 ± 0.06 | 0.81 ± 0.05 | <0.001 | 0.84 ± 0.05 | 0.83 ± 0.05 | 0.83 ± 0.06 | 0.82± 0.05 | 0.002 |
| SII | 557.86 ± 261.63 | 514.24 ± 277.22 | 497.63 ± 262.14 | 412.77 ± 177.12 | <0.001 | 562.08 ± 280.85 | 513.02 ± 240.18 | 478.90 ± 266.41 | 430.12 ± 201.14 | <0.001 |
| SIRI | 1.11 ± 0.57 | 1.04 ± 0.63 | 1.23 ± 0.71 | 1.06 ± 0.61 | 0.052 | 1.16 ± 0.67 | 1.09 ± 0.58 | 1.11 ± 0.64 | 1.09 ± 0.65 | 0.793 |
| PIV | 293.73 ± 164.70 | 267.63 ± 188.39 | 298.39 ± 207.32 | 232.43 ± 143.27 | 0.007 | 302.70 ± 186.48 | 277.28 ± 170.61 | 260.75 ± 169.31 | 252.16 ± 186.90 | 0.097 |

**
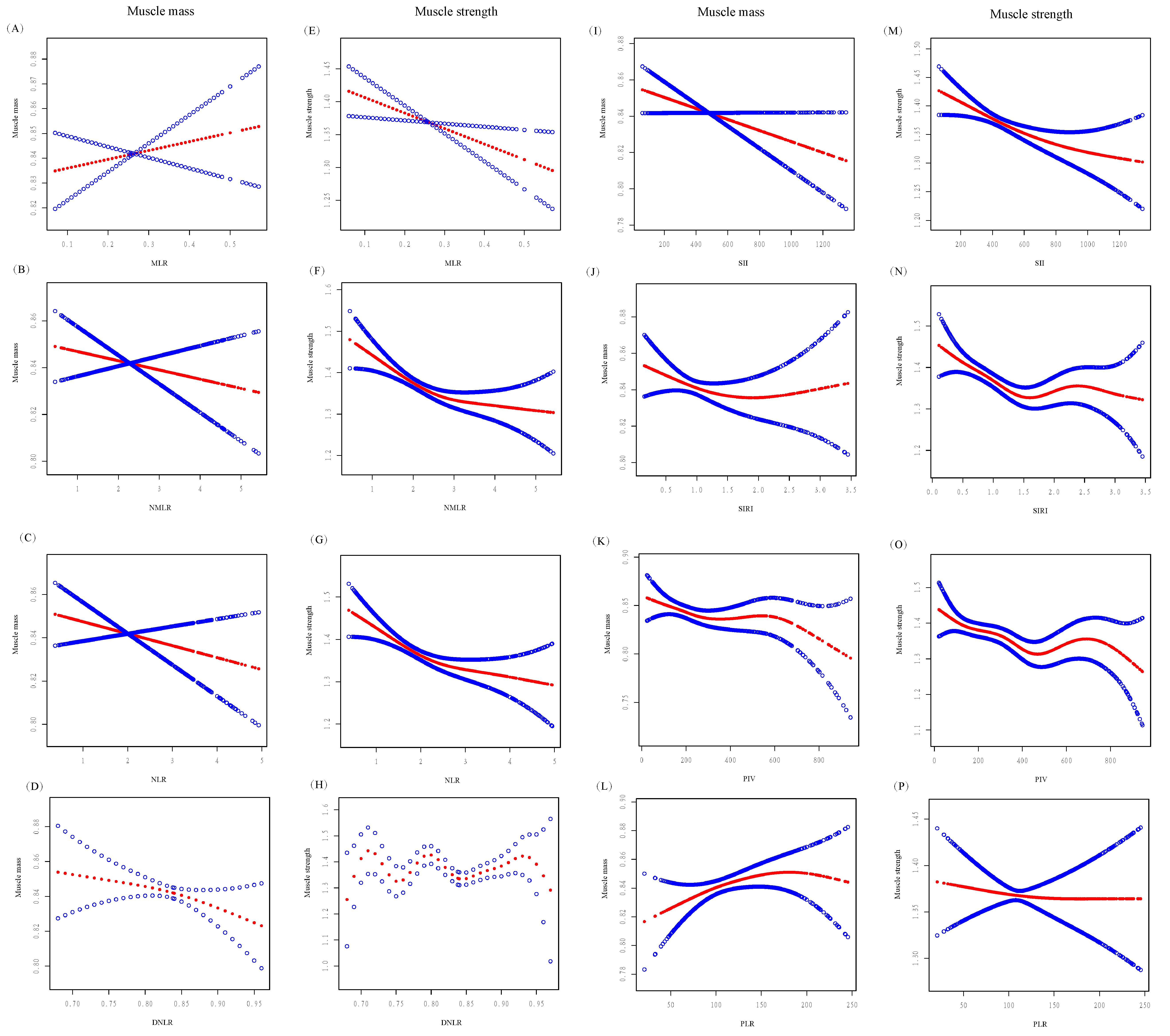
Supplementary Figure 1.** Smooth curve fitting of the eight inflammatory indicators with muscle mass or strength. Two blue curves representing the 95% confidence interval of the results.


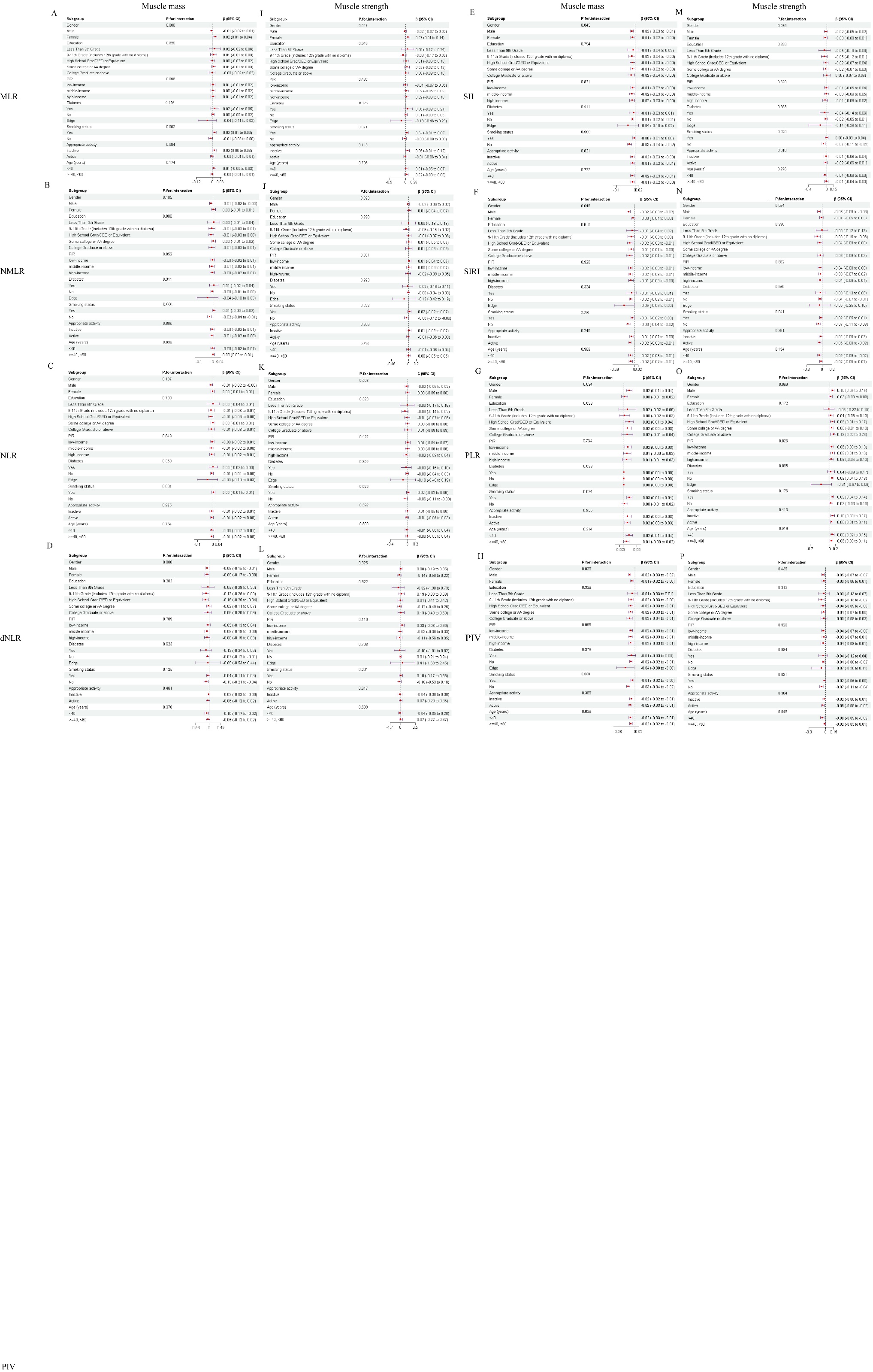
**Supplementary Figure 2.** Subgroup analyses of the associations of eight inflammatory markers with muscle mass and strength were performed.


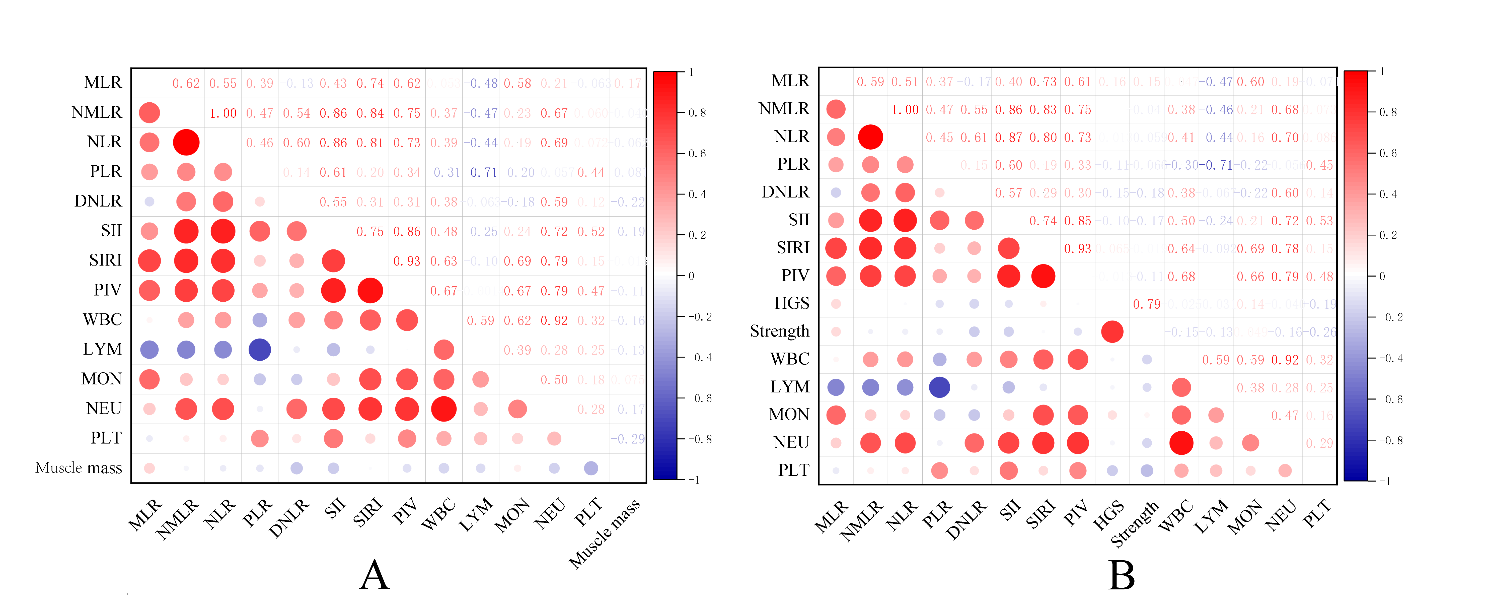
**Supplementary Figure 3.** Spearman's analysis. Correlation coefficients between complete blood cell parameters and their associations with sarcopenia and LMS were compared using Spearman's analysis.


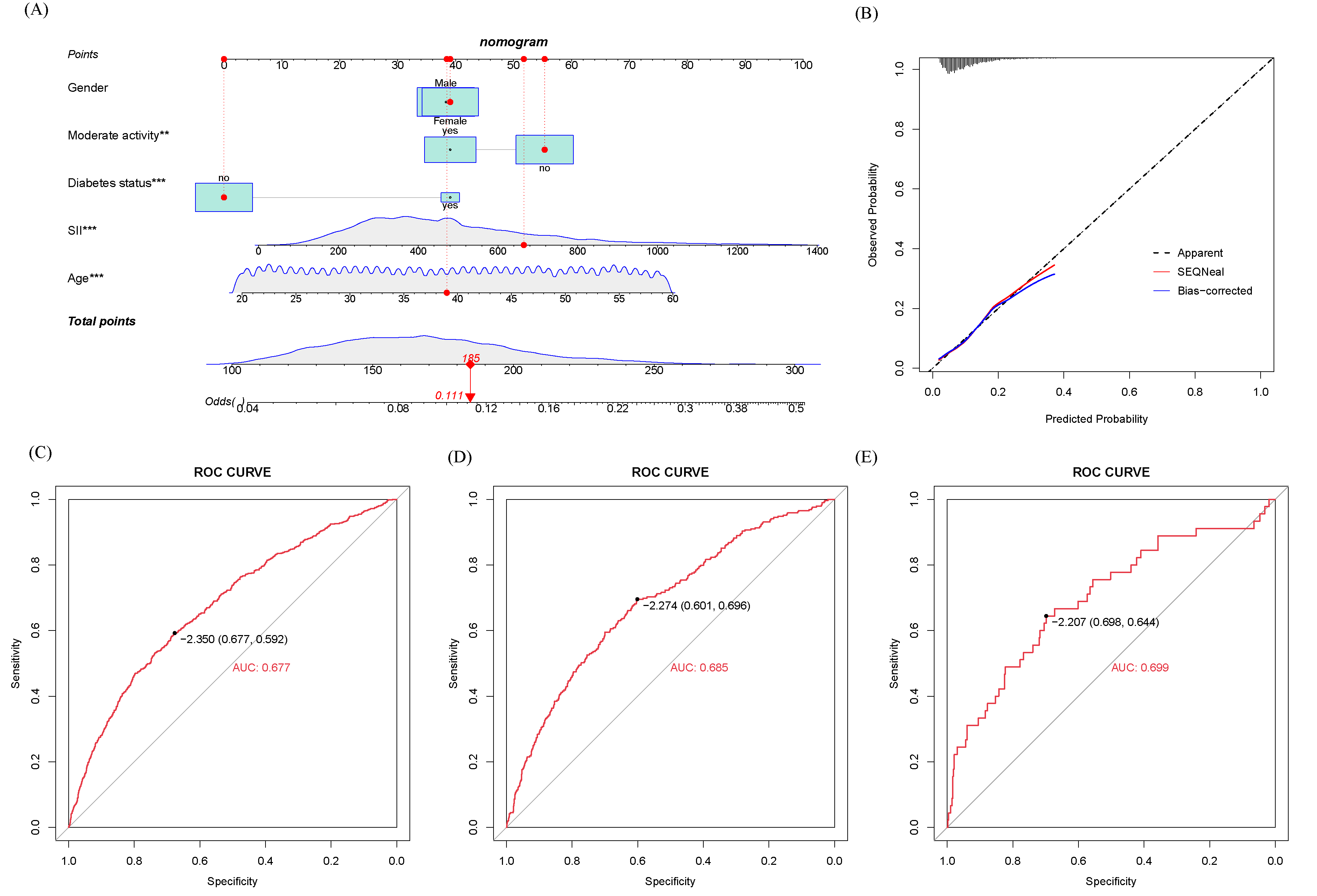


**Supplementary Figure 4.** Nomogram and model validation for predicting low muscle mass based on SII and related factors.(A) shows the nomogram constructed based on SII and related factors for predicting low muscle mass.(B) represents the calibration curve of the model in the external validation dataset, demonstrating the agreement between predicted probabilities and actual observations.(C-E) illustrate the ROC curves for the training set, validation set, and external validation set, respectively, highlighting the model's predictive performance and discriminatory ability across different datasets. Note:“*” indicates a p-value of <0.1 in both univariable and multivariable regression models, “**” indicates a p-value of <0.01, and “***” indicates a p-value of <0.001 in both models.

**
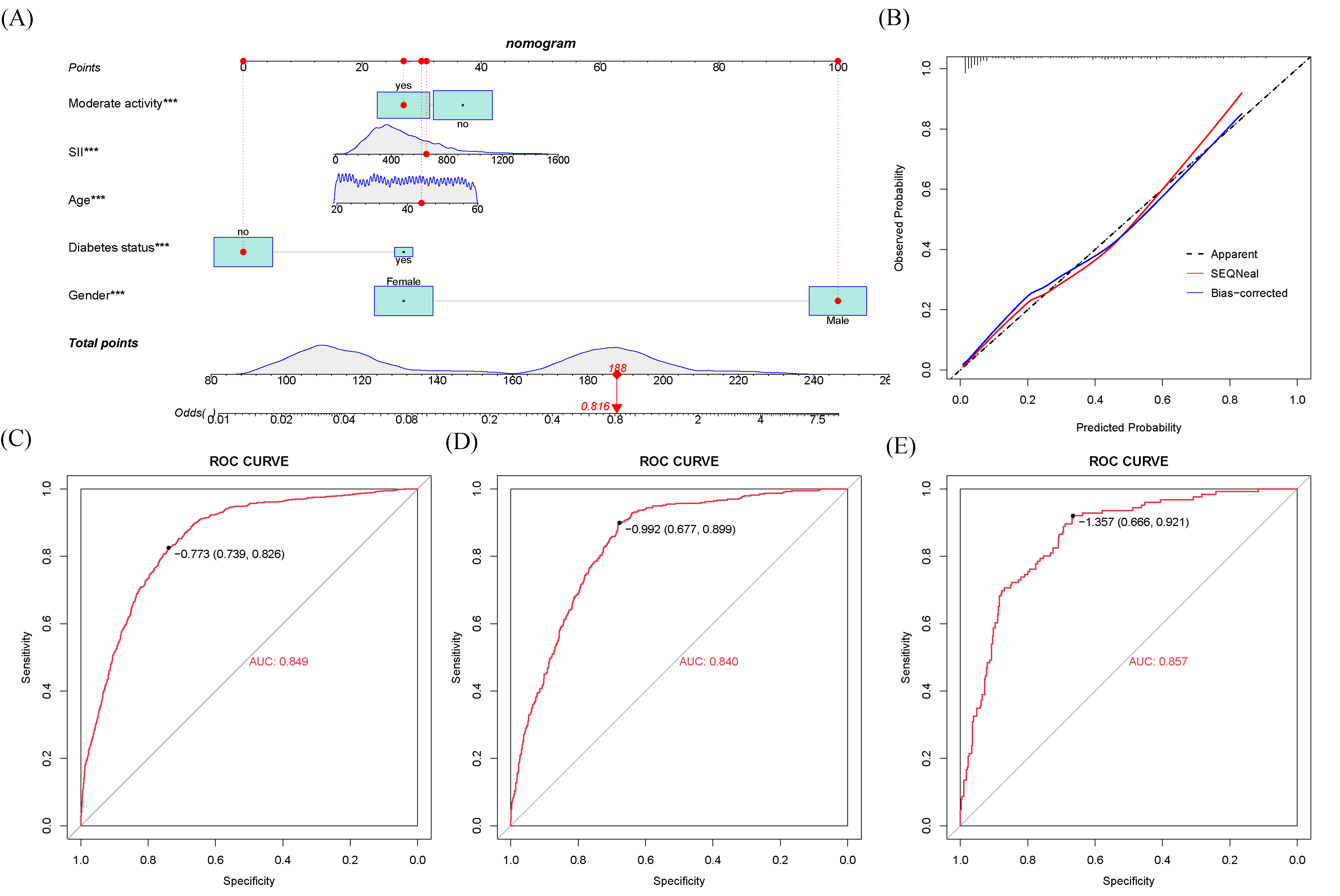
**

**Supplementary Figure 5.** Nomogram and model validation for predicting low muscle strength based on SII and related factors.(A) shows the nomogram constructed based on SII and related factors for predicting low muscle strength.(B) represents the calibration curve of the model in the external validation dataset, demonstrating the agreement between predicted probabilities and actual observations. (C-E) illustrate the ROC curves for the training set, validation set, and external validation set, respectively, highlighting the model's predictive performance and discriminatory ability across different datasets. Note:“*” indicates a p-value of <0.1 in both univariable and multivariable regression models, “**” indicates a p-value of <0.01, and “***” indicates a p-value of <0.001 in both models.


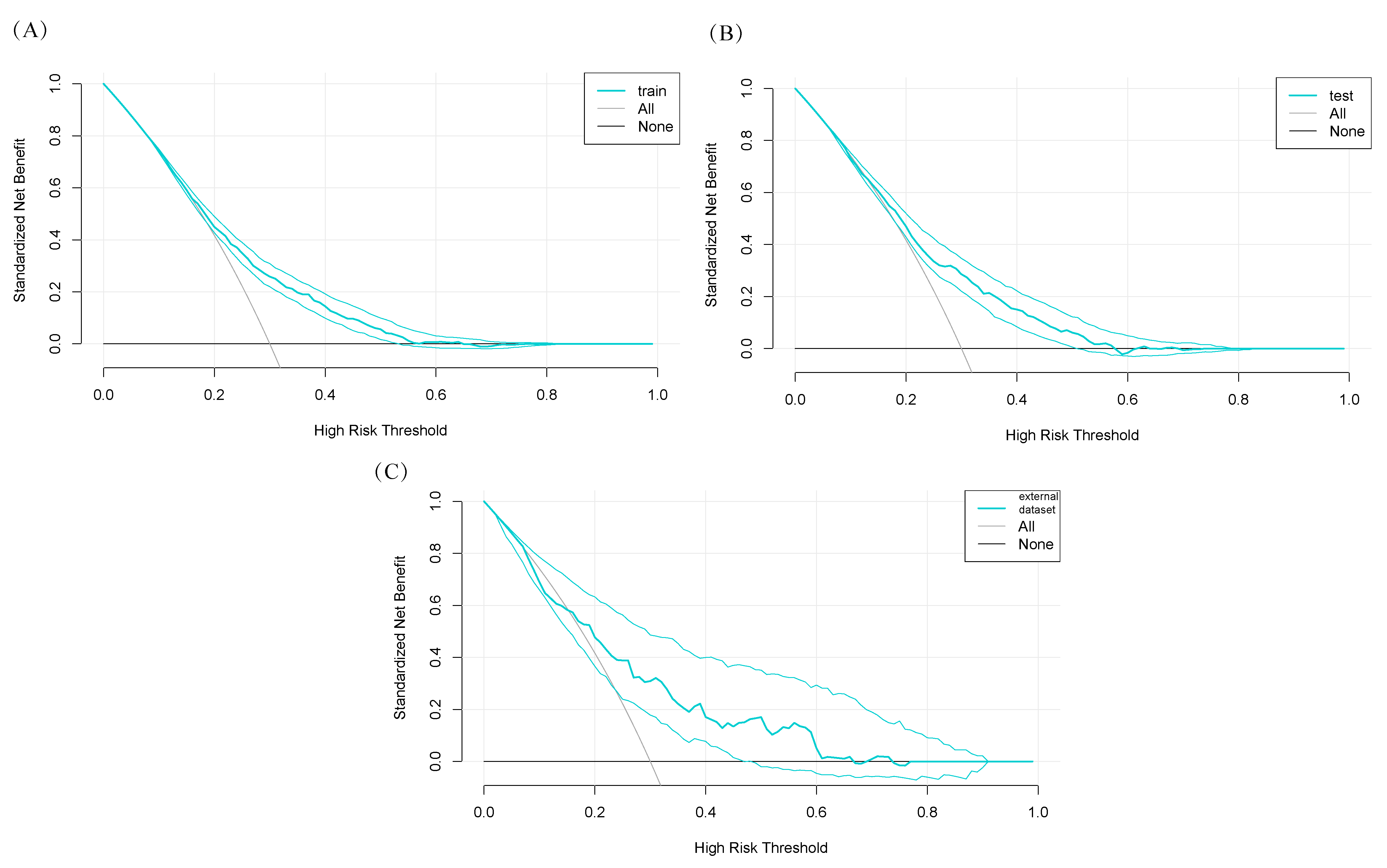


**Supplementary Figure 6.** Decision curve analysis for evaluating the clinical utility of the model in predicting low muscle mass.(A) represents the decision curve for the training set, illustrating the net benefit of the model across different high-risk thresholds;(B) represents the decision curve for the validation set, assessing the clinical utility of the model in the validation dataset;(C) represents the decision curve for the external validation set, evaluating the model's predictive performance and practical value in an external dataset.


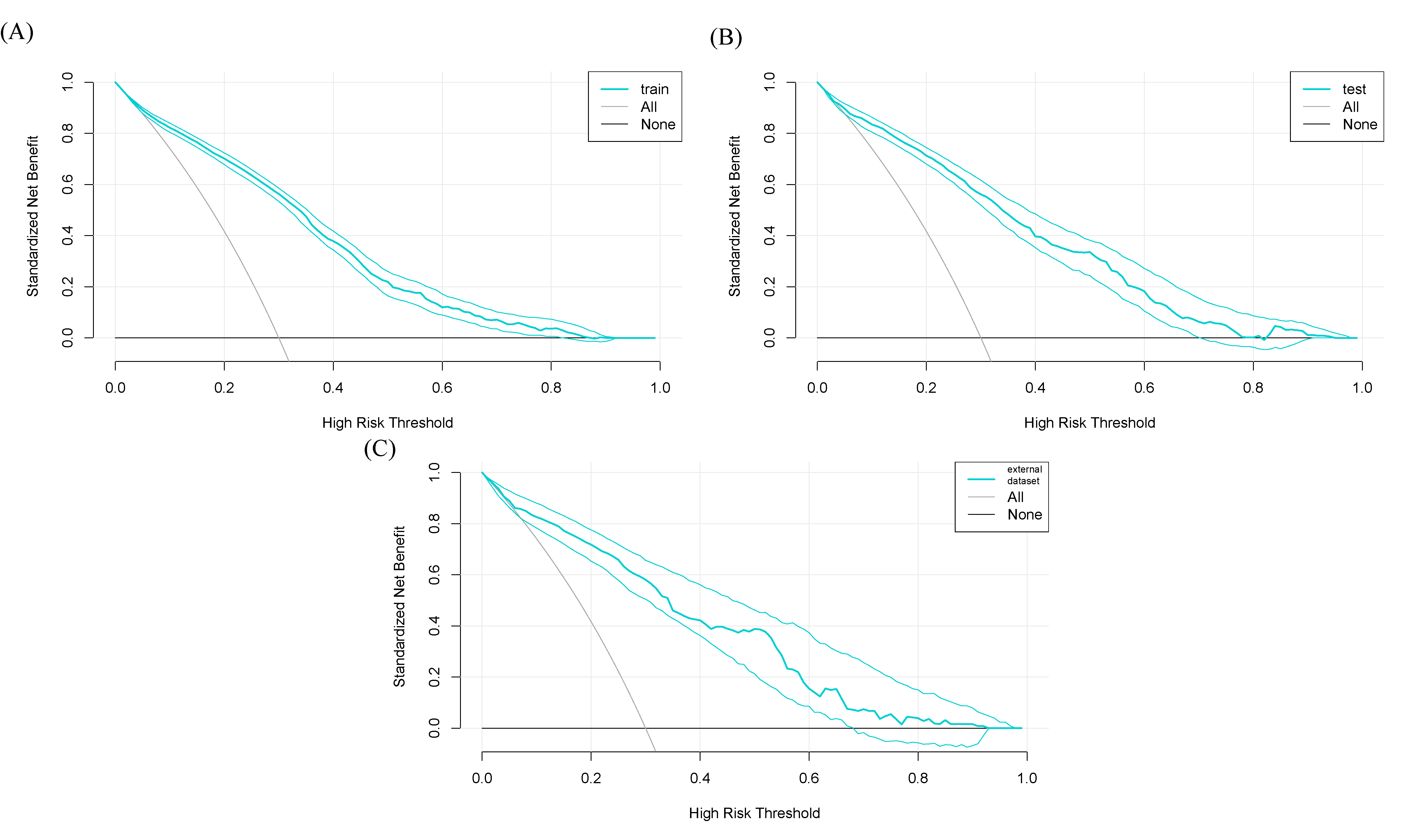


**Supplementary Figure 7.** Decision curve analysis for evaluating the clinical utility of the model in predicting low muscle strength.(A) represents the decision curve for the training set, illustrating the net benefit of the model across different high-risk thresholds;(B) represents the decision curve for the validation set, assessing the clinical utility of the model in the validation dataset;(C) represents the decision curve for the external validation set, evaluating the model's predictive performance and practical value in an external dataset.
